# The history of neuromyelitis optica. Part 2: ‘Spinal amaurosis’, or how it all began

**DOI:** 10.1186/s12974-019-1594-1

**Published:** 2019-12-28

**Authors:** S. Jarius, B. Wildemann

**Affiliations:** Department of Neurology, Molecular Neuroimmunology Group, University of Heidelberg, Otto Meyerhof Center, Im Neuenheimer Feld 350, 69120 Heidelberg, Germany

**Keywords:** Neuromyelitis optica (NMO), Neuromyelitis optica spectrum disorder (NMOSD), Devic’s syndrome, Optic neuritis, Transverse myelitis, Multiple sclerosis, Spinal amaurosis, Amaurose spinale, Aquaporin-4 (AQP4) antibodies, Myelin oligodendrocyte glycoprotein (MOG) antibodies, History of medicine, History of neurology

## Abstract

Neuromyelitis optica (NMO) was long considered a clinical variant of multiple sclerosis (MS). However, the discovery of a novel and pathogenic anti-astrocytic serum autoantibody targeting aquaporin-4 (termed NMO-IgG or AQP4-Ab), the most abundant water channel protein in the central nervous system, led to the recognition of NMO as a distinct disease entity in its own right and generated strong and persisting interest in the condition. NMO is now studied as a prototypic autoimmune disorder, which differs from MS in terms of immunopathogenesis, clinicoradiological presentation, optimum treatment, and prognosis. While the history of classic MS has been extensively studied, relatively little is known about the history of NMO. In Part 1 of this series we focused on the late 19th century, when the term ‘neuromyelitis optica’ was first coined, traced the term’s origins and followed its meandering evolution throughout the 20th and into the 21st century. Here, in Part 2, we demonstrate that the peculiar concurrence of acute optic nerve and spinal cord affliction characteristic for NMO caught the attention of physicians much earlier than previously thought by re-presenting a number of very early cases of possible NMO that date back to the late 18th and early 19th century. In addition, we comprehensively discuss the pioneering concept of ‘spinal amaurosis’, which was introduced into the medical literature by ophthalmologists in the first half of the 19th century.

## Background

Neuromyelitis optica (NMO, Devic’s syndrome) is a rare syndrome characterised by optic neuritis and myelitis [1]. For many decades, NMO was considered a clinical subtype of multiple sclerosis (MS). However, the discovery of pathogenic autoantibodies to aquaporin-4 (AQP4) in patients with NMO, which are absent in MS, led to the recognition of NMO as a disease entity in its own right [2–4] and generated strong and persisting interest in the syndrome. While the history of MS has been extensively studied, relatively little is known about the early history of NMO [5].

In Part 1 of this series of articles [5], we focused on the late 19th century, when the term ‘neuromyelitis optica’ was first coined. We traced the origins of this peculiar term in the 19th century French-, English-, and German-language literature and followed its definition’s meandering evolution throughout the 20th and into the 21st century.

Here, in Part 2, we turn the spotlight onto the very beginnings of the recognition of NMO as a distinct syndrome and re-present and discuss a number of early 19th century reports on cases of possible NMO, all of which precede Eugéne Devic (1858–1930) and Fernand Gault’s (1873–1936) disease-defining work on NMO, which appeared in 1894, by years or even decades, among them a detailed clinical case description and an early post-mortem analysis. These early reports demonstrate that the rare and intriguing coincidence of acute spinal cord and optic nerve damage characteristic for NMO had caught the attention of the medical world much earlier than previously thought.

At that time, the syndrome still went under another name: that of ‘*amaurose spinal*’ (or ‘*spinal amaurosis*’ in English and ‘*Spinale Amaurose*’ in German). That term had been introduced by French ophthalmologists in the early 19th century to refer to the unusual coincidence of spinal cord and optic nerve disease. The term remained in use throughout the entire 19th century but fell into oblivion after the turn of the century, when ‘neuromyelitis optica’, the designation proposed by Devic and Gault in 1894, increasingly prevailed. With that change in nomenclature, awareness of the long history of NMO as a subject of study in ophthalmology declined rapidly, and most of the early observations of NMO reported as ‘spinal amaurosis’ re-presented here have been almost completely forgotten.

## Emergence of a novel concept

### Julius Sichel and the concept of ‘amaurose spinal’ (1832/1837)

**Fig. 1:**
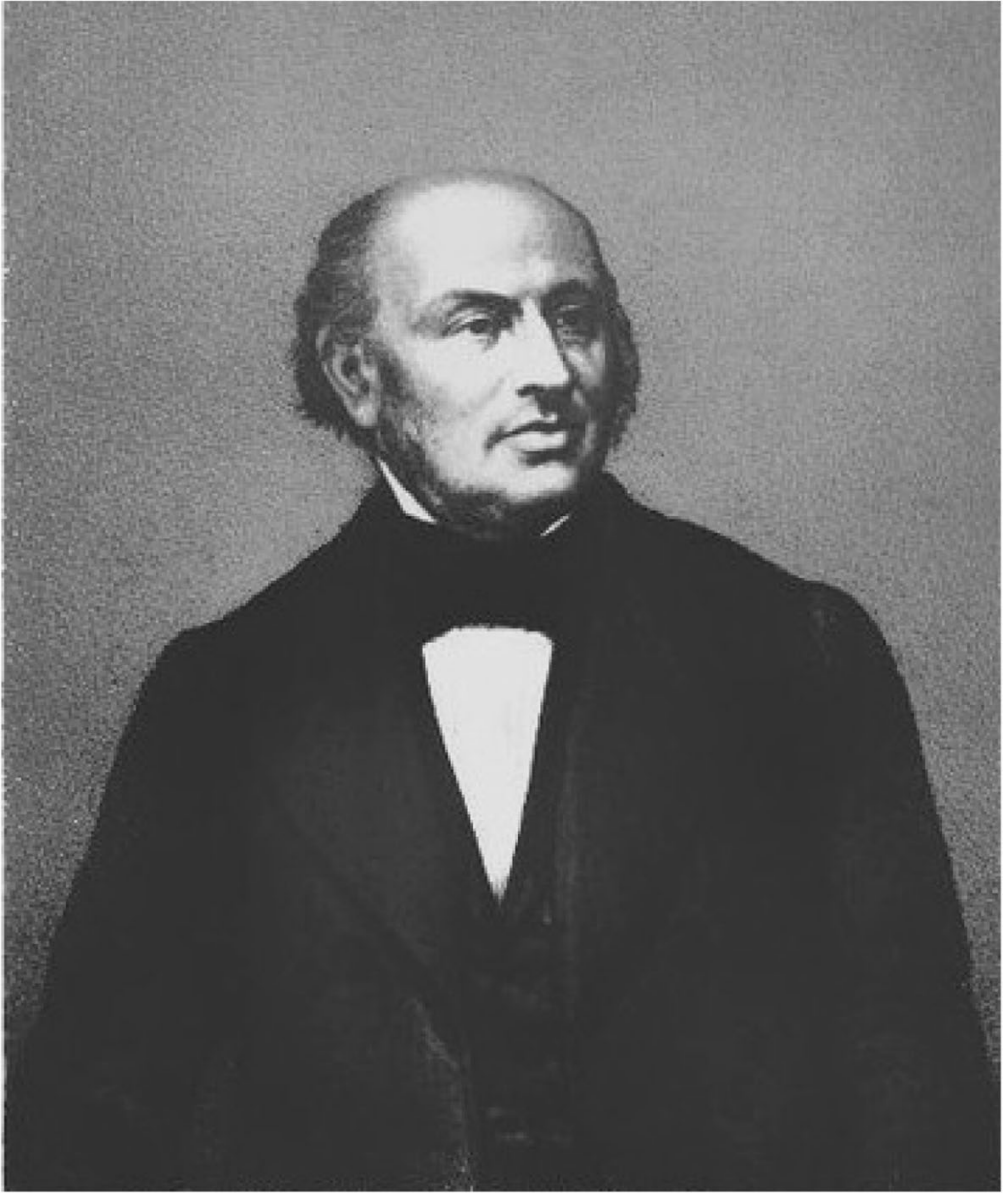
Frédéric Jules Sichel (1802–1868)

The years following the French revolution saw a dramatic decline in the field of ophthalmology in France. In 1846, Phillip Franz von Walther (1782–1849), one of the founding fathers of scientific surgery and ophthalmology in Germany, a pupil of Georg Joseph Beer (1763–1821) and co-editor (together with Albrecht von Graefe [1828–1870]) of the *Journal für Chirurgie und Augenheilkunde*, could write: “*Unbegreiflich ist, wie in einem Lande, in welchem früher die ersten Anfänge einer rationellen und wissenschaftlichen Begründung der Augenheilkunde durch Maître-Jan, St. Yves, Méjan, Daviel, J.L. Petit u. a. sich zeigten, mit dem Eintritt der Revolution der Faden der Entwicklung so ganz abreißen und für mehrere Dezennien totale Verdunklung entstehen konnte” [It is beyond comprehension how, in a country in which earlier the beginnings of a rationale and scientific foundation of ophthalmology were laid by Maître-Jan, St. Yves, Méjan, Daviel, J. L. Petit and others, the thread of development could break so completely and total obfuscation emerge]* [6]. Von Walther’s harsh verdict was shared by many outside France, as carefully documented by Julius Hirschberg (1843–1925) [7]. When ophthalmology was revived in France in the 1830s and 1840s, the renaissance was mainly driven by a number of migrants, among whom Julius Sichel (1802–1886) must be considered the most important figure. Sichel (Fig. 1) was a pupil of Johann Lukas Schönlein (1793–1864) – one of the main modernisers of medicine in Germany (today best remembered for coining the terms ‘tuberculosis’ and ‘haemophilia’, as well as for the description of Schönlein-Henoch’s purpura) and teacher of Rudolf Virchow (1821–1902) – and of Christoph Friedrich Jäger (1784–1871), a former assistant to Beer. Sichel went to Paris in 1830 and founded one of the first French ophthalmological policlinics (‘*dispensaire*’) there in 1833. In 1834, he was made a French citizen. In 1837, he published his *Traité de l’ophthalmie, la cataracte et l’amaurose* [8], which soon became one of the most influential textbooks in its field.

It was in that work that Sichel introduced the concept of *‘amaurose spinale’ [spinal amaurosis]*, proposing that amaurosis may be linked to spinal cord disease in some cases: *“L’amaurose peut également être symptomatique d’une affection de la moelle épinière ( …*) *Mais ce qui constitue le caractère principal de cette espèce d’amaurose, c’est là concomitance de symptômes qui se rapportent particulièrement à une affection co-existante de l’axe spinal ou d’une partie du cordon de la moelle épinière” [Amaurosis can also be a symptom of a disease of the spinal cord (...) What constitutes the main character of this type of amaurosis is the concomitant presence of symptoms that particularly indicate co-existing affection of the spinal axis or of a part of the spinal cord].*

Sichel dedicated a whole chapter (entitled “*Genre VI. — Amaurose spinale*”) to the topic, in which he points to the importance of proper neurological examination in patients presenting with amaurosis: *“Ces détails sur l’exploration de la moelle épinière sont d’autant plus importants, que de l’exactitude avec laquelle l’examen de la colonne est fait dépend la certitude du diagnostic, et la possibilité de confirmer par de nouveaux faits l’existence d’une connexion étroite entre les affections de la moelle épinière et certaines espèces d’amaurose” [The details of the exploration of the spinal cord are all the more important as the accuracy of the spinal examination determines the certainty of the diagnosis and the possibility of confirming by new findings the existence of a close connection between the affections of the spinal cord and of certain types of amaurosis]* [8]. Showing profound understanding of spinal cord function, he recommended physicians should pay attention in such patients not only to paralysis and numbness of the upper and lower limbs, but also to symptoms such as respiratory distress, pain, girdle-like dysesthesia (“*engourdissements*”), jerks and involuntary contractions of the extremities, convulsions, arrhythmia, circulatory problems, incontinence, urinary retention or constipation, impotence, and amenorrhoea. He even pointed to the potential occurrence of brainstem symptoms such as dysphagia and oculomotor disturbances: *“Quelquefois il s’y joint des symptômes d’un dérangement dans la motilité du globe oculaire, diplopie” [Sometimes there are symptoms indicating disturbance in motility of the globe of the eye, diplopia]*. Brainstem involvement has been recently rediscovered as a frequent feature of both AQP4-immunoglobulin G (IgG) and myelin oligodendrocyte glycoprotein (MOG)-IgG-positive NMO [1, 9, 10], and respiratory insufficiency due to brainstem or upper cervical cord lesions is nowadays the most common cause of death from NMO [11]. Similarly, tonic brainstem attacks have been shown to occur frequently in AQP4-IgG-positive NMO, and seizures have been observed both in AQP4-IgG-positive and in MOG-IgG-positive NMO in recent studies [12–19].

Regarding the question of how spinal cord disease and amaurosis could be related, Sichel could only speculate. He referred to the network-like organisation of the nervous system and posited a direct link between the eye and the spinal cord: *“La continuité de toutes les parties du système nerveux, et les rapports directs de liaison entre l’appareil nerveux de l’organe de la vision et l’axe spinal, sont là pour expliquer l’origine des amauroses de cette espèce” [The continuity of all parts of the nervous system, and the direct connections between the nervous apparatus of the organ of vision and the spinal axis, explain the origin of this sort of amaurosis]*.

In accordance with contemporary ideas on disease pathogenesis, he distinguished irritative (*“congestive ou éréthique nerveuse”*) and torpid cases of spinal amaurosis. Among the torpid cases he counted, inter alia, those occurring during excessive breast feeding *“bei nervösen und reizbaren Frauen” [in nervous and irritable women]*. Interestingly, recent studies indeed suggest an increase in disease activity in NMO during the first year post partum [20, 21]). Among the irritative cases he counted, besides cervical injuries and many others, cases caused by toxins, but frankly conceded: *“Nous ne possédons que fort peu de données sur cette variété de l’amaurose spinale” [We have very little in the way of data on this variety of spinal amaurosis]*. However, he strongly believed that besides these conditions, which he classified as ‘functional’, also ‘organic’ diseases of the spinal cord, which acted by *“inflammation ou congestion”*, could cause amaurosis and recommended *“recueillir avec un soin scrupuleux tous les matériaux propres à jeter du jour sur ce sujet” [to collect with scrupulous care all the proper materials to throw light on this subject]*.

Whether Sichel personally attended a patient with true NMO is unclear. In his *Traité de l’amaurose ou de la goutte-sereine*, Charles Deval (1806–1862), an ophthalmologist and former student of Sichel, later recollected the case of a young woman with partial visual loss, in whom Sichel (in Deval’s presence) diagnosed “*sub-inflammation de la moelle épinière*” *[subinflammation of the spinal cord]* and whom he treated with leeches [22]. In his own writings, Sichel makes reference to a 14-year-old young girl whom he had seen in 1832. This girl presented with bilateral visual deficiency with (transiently) dilated pupils associated with spinal pain and contractions of the extremities. Complete remission was achieved following *“un traitement révulsif, joint aux antispasmodiques, à la valériane, etc.” [revulsive treatment, combined with antispasmodics, valerian,* etc.*]* [8]. Whether the two reports relate to the same patient remains unknown.

Sichel was well aware of the obvious limitations of his recommendations, which he could not found on the authority of previous authors, but was convinced about the potential clinical relevance of his observations: *“Nous sentons vivement le besoin de nouveaux éclaircissements sur ce point obscur de la pathologie oculaire que nous osons seulement effleurer dans l’intention d’y appeler l’attention des praticiens” [We feel strongly the need for further clarification on this obscure point of ocular pathology that we only dare to touch with the intention of calling it to the attention of practitioners]* [8]. This call would be answered sooner than Sichel may have expected.

### Carron du Villards (1838): “Myelitis and complete amaurosis”

**Fig. 2:**
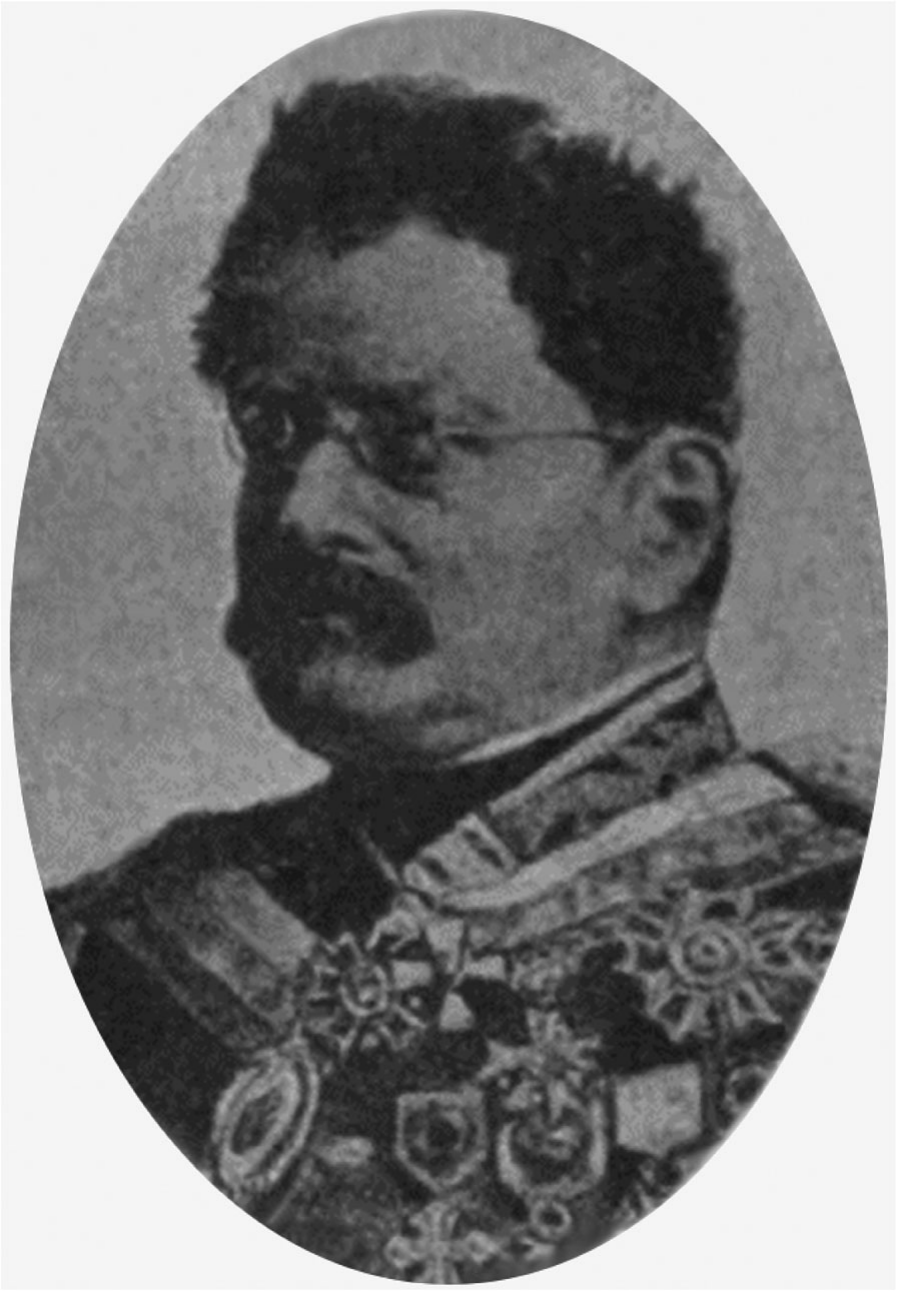
Charles Joseph Frédéric Carron du Villards (1801–1860)

Besides Sichel, it was Charles Joseph Frédéric Carron du Villards (1799?-1860) (Fig. 2), who would cast some light into the ‘darkness’ described by von Walther. Carron du Villards studied with Antonio Scarpa (1752–1832) – the famous Pavian surgeon and anatomist, first physician to Napoleon I, pupil of Giovanni Battista Morgagni (1682–1771), one of the founding fathers of pathological anatomy, author of the first ophthalmological textbook in the Italian language [23], and once characterised as “*the highest culmination of the Galenic tradition of ophthalmology*” [24] – and then moved to Paris in 1828, where he took part in Jacques Lisfranc’s (1790–1847) operation courses and later, together with Salvatore Furnari (1808–1866), founded his own ophthalmological *dispensaire* [7]. Being originally a native of Savoy, he was naturalised in 1832 and would later describe himself as an “*français d’adoption*” [25] (which did not restrain him from sharing von Walther’s pessimistic perspective of contemporary French ophthalmology, complaining in 1838: “*Pourquoi depuis 1799, la France a-t-elle cessé de payer son tribut à l’edifice de la science ophthalmologique?” [Why did France after 1799 stop paying its tribute to the construction of scientific ophthalmology?]* [26]). However, he never settled in one place for long. In 1888, 28 years after his death, an obituary appeared in the *Annales d’Oculistique* [27], which, along with Hirschberg’s notes, allows us to retrace the many stations of his restless and adventurous life: 1841 found him in Amsterdam, 1843 in Luxembourg, and his subsequent travels took him to Norway, Greece, Sphacteria, Candia, Tripoli, Tangiers, Liberia, Sierra Leone, Havana (where he worked for 2 years and survived two invasions), Puerto Rico, Mexico, Chile, Peru and Venezuela [7, 28, 29]. In Mexico, he bore the title of a general in the medical service; three times he was a castaway, and once he was shot and wounded [7]. In 1858, Carron du Villards arrived in Rio de Janeiro, where – under imperial protection – he would found the first public ophthalmological hospital, and where he was to die in 1860.

Carron du Villards was a prolific writer, whose *Guide pratique pour l’etude et le traitement des maladies des yeux* (Brussels, 1838) [25] is considered one of the most important ophthalmological textbooks of the period [7, 30].

It is in this textbook that Carron du Villards speaks about *“[une] espèce d’amaurose ( …*) *qui est sympathique d’une affection aiguë ou chronique de la moelle épinière” [a type of amaurosis (…*) *that is sympathetic to an acute or chronic affection of the spinal cord]* and, importantly, declares, “*j’ai vu plusieurs individus atteints de myélite qui furent complètement amaurotiques*, *pendant toute la durée de la maladie, la cécité disparut en même temps que l’inflammation intra-vertébrale” [I saw several individuals with myelitis who were completely amaurotic throughout the duration of the disease; the blindness disappeared at the same time as the intravertebral inflammation]*. Like Sichel, he refers to the coincidence of optic nerve and spinal cord disease by using the term ‘*amaurose spinale*’.

Based on his observations, he, just like Sichel before him, points to the need of carefully examining patients with amaurosis for signs of spinal cord inflammation, recommending, “*il faut examiner avec soin la colonne vertébrale et promener sur tout son trajet des corps très-chauds ou très-froids, qui déterminent presque toujours une douleur ou tout au moins une sensation désagréable dans le point malade* “*[the vertebral column must be carefully examined, and very hot or very cold objects, which almost always generate pain - or at least an unpleasant sensation - at the site of disease, must be moved all the way along the spine]* [25].

Regarding therapy, he suggests bloodletting, which was considered to have “antiphlogistic” effects by some contemporary authors [31], and the application of leeches along the spine, in addition to other remedies of the time such as cupping, blistering and moxa. The former recommendation is interesting in the light of recent studies demonstrating that plasma exchange is effective in patients with acute NMO [32–35]. Given the enormous amounts of blood extracted by physicians performing phlebotomy in the 19th century (up to 2000 cc within the first 24–48 h, greatly overestimating the total blood volume, or until the patient fainted) [36], it is tantalising to speculate as to whether repeated bloodletting might indeed have had some therapeutic effect [31].

We carefully perused Scarpa’s works on ophthalmological topics. However, it seems that Carron du Villards’ teacher never made any mention of spinal cord diseases as a cause of amaurosis in his writings.

### Pétrequin (1841): an early post-mortem report

**Fig. 3:**
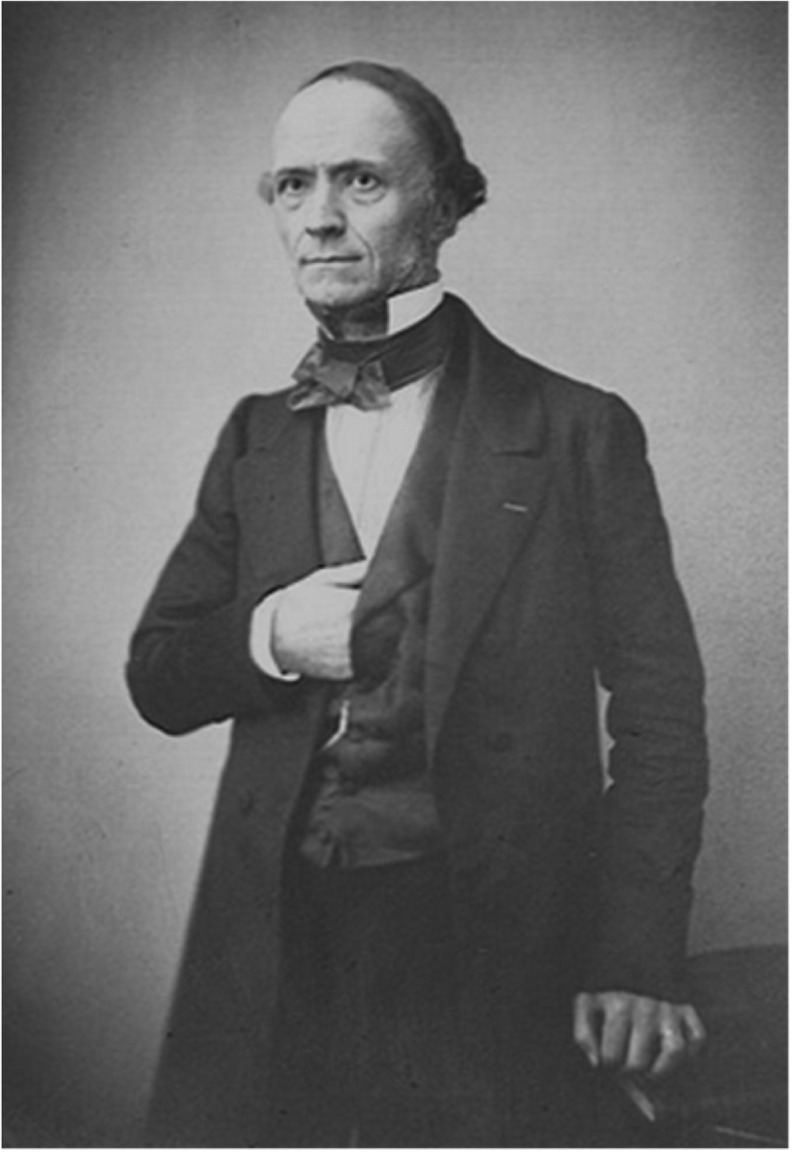
Joseph Pierre Eléonor Pétrequin (1809–1876)

Carron Du Villards’ original works are numerous (a comprehensive list can be found in Hirschberg’s *Geschichte der Augenheilkunde* [7], volume XIV, 3, §568). Among them is a short letter to a certain Joseph Pierre Eléonor(d) Pétrequin (1809–1876; see [37] for a biographical sketch) (Fig. 3) which appeared in the first volume of the *Annales d’Oculistique et de Gynécologie* [25] and marked the beginning of a debate about the best method for surgical treatment of cataract [25, 38–40]. It was the same Pétrequin, a then famous surgeon at the Hôtel-Dieu in Lyon with a profound interest in the history of medicine [41, 42], who would shortly afterwards publish a further case of what Sichel and Carron du Villards had described as ‘*amaurose spinale*’. Pétrequin’s report is to be found as observation 24 (entitled *“Amaurose dyscrasique double, passée à l’état torpide complet, et compliquée de paraplégie. Guérison de l’amaurose”*) in his *Traité pratique de l’amaurose ou goutte-sereine* (1841) [43].

The patient, a 19-year-old male weaver (“*lymphatique et scrophuleux quoique robuste*” *[lymphatic and scrofulous but robust]*) from St.-Joux (Rhóne) experienced rapid-onset bilateral visual loss in May 1838, accompanied by continuous orbital pain. When the patient first presented on 3rd of June, Pétrequin found him completely blind with an amaurotic facies; the pupils were dilated and insensible to light. Pétrequin started to treat his patient with bloodletting and leeches, and, in addition, with purgatives (eau de Sedlitz, jalap, calomel, aloe) and a blister in the neck (as a counter-irritant). These measures were followed by an increase in vision and amelioration of the patient’s headache. However, on 22nd of June the young man developed lumbar pain, dysaesthesia of the legs and urinary retention. Shortly afterwards, he developed severe paraparesis (plegia of the right leg; capable of elevating the left leg from the bed). At the same time, his sight continued to improve: by 4th of July he was able to count fingers and recognise bright colours, and by 11th of July, after seven frictions with a nux vomica tincture, vision had further increased to a degree that he was able to recognise individuals at eight steps’ distance. On 18th of July, after continued treatment with nux vomica frictions, he was able to decipher large letters. By contrast, the paralysis continued and, 1 week later decubitus at the trochanters and the sacrum as well as urinary incontinence were noted, and the patient “*s’affaiblit et dépérit peu à peu*”; “*le malade n’offre pas d’espoir” [weakens and decays little by little; the patient does not give reason to hope]*. Over the next few days the decubital ulcers became worse (“*disséquent tout le basin*”), and the patient subsequently died on 2nd of September.

Besides respiratory insufficiency, sepsis from decubital ulcers and/or urinary retention was one of the most common causes of death from NMO well into the twentieth century, resulting in many patients not surviving their first relapse. This – and the lack of long-term data in many other cases [44] – may explain why NMO was long considered to be a mostly monophasic disease. Mortality has dramatically declined in recent years with routine use of high-dose steroid treatment, plasma exchange and immunosuppressants (in particular B cell-depleting agents) [1, 11], and with more and more patients surviving the first attack, NMO was found to be a relapsing disease in the vast majority of cases, especially in AQP4-IgG-seropositive patients [1].

What makes Pétrequin’s report particularly valuable is the fact that it is accompanied by a detailed post-mortem report. While the pathologist found the brain, the cerebellum, and the spinal meninges unremarkable, he noted brownish-grey discoloration and softening of the spinal marrow beginning at the level of the 10th dorsal nerve, which he classified as the remnants of myelitis. The retina and optic nerves were considered healthy except for capillary congestion. The latter finding, although it may well reflect the restricted diagnostic means of the time (apparently, no microscopic analysis was performed), is in accordance with the patient’s visual improvement as already appreciated by Pétrequin: “*aucune lésion profonde ne formait obstacle à la guérison*” *[no profound lesion was present as an obstacle to healing]*. Current studies have found complete recovery from optic neuritis (ON) in as many as 33% of attacks in NMO [1].

Pétrequin’s report was made soon made known to an audience outside France by being re-printed in the second volume of the *Annales de la Société de Sciences Naturelles, de Bruges* [45].

Of note, two further brief reports on patients with paresis and visual disturbance can be found elsewhere in Pétrequin’s writings [46]: A 26-year-old woman whose left eye had been *“sujet à des ophthalmies”* for a long time was admitted to the Hôtel-Dieu in Lyon in 1828 with paresis of all extremities and urinary retention requiring catheterisation; she recovered from paresis after 4 years. A 55-year-old woman developed transient amaurosis and hemiplegia in 1833 and subsequently completely recovered (following treatment with an alcoholic extract of nux vomica).

Importantly, Pétrequin believed his patients’ symptoms to have been caused by what he called – using an originally Galenic term – dyscrasia (*“amaurose dyscrasique”*), i.e. an abnormal, pathological composition of the blood. This is highly interesting in the light of the fact that most later authors would attribute ON in patients with spinal cord disease either to damage to the spinal origin of the sympathetic nerve (and, in consequence, nutritional damage to the optic nerve as a result of disturbed blood supply) or to ascending myelitis – and thus to an anatomic or at least spatial connection between the two remote sites. It would take until 1896 for the two latter theories to be challenged by Karl Katz (1869–1944), a doctoral student to Theodor Karl Gustav von Leber (1840–1914) in Heidelberg, who concluded: *„die Gleichartigkeit der pathologisch-histologischen Veränderungen [weist] mit Notwendigkeit auf eine gemeinschaftliche im Blut circulierende Noxe hin* “*[the similarity of the pathohistological changes [indicates] necessarily a shared noxa circulating in the blood]*, and another century for two such ‘noxae’ – pathogenic autoantibodies to AQP4, a water channel protein abundantly expressed in the central nervous system (CNS) [2, 3, 47–49], and pathogenic autoantibodies to MOG [10, 50–55] – to be identified in the serum of patients with NMO.

In pondering about some sort of dyscrasia underlying ‘spinal amaurosis’, Pétrequin – knowingly or unknowingly – possibly followed Konrad Johann Martin Langenbeck (1776–1851), who had stated already in 1818 in his *Reflexionen über die Natur, Ursachen und Heilung des schwarzen Stars*: *“Gehen wir auf die Veranlassungen zurück, so finden wir in so vielen Fällen die Amaurosis offenbar als Folge mancherley Dyscrasien, deren erste Wirkung Entzündung ist” [regarding causation, we find that in many cases amaurosis is plainly the result of dyscrasias, the first effect of which is inflammation]* and – himself drawing a parallel to humoral pathology – discussed that *“Ablagerungen irgendeines Krankheitsstoffes”* *[deposits of some sort of pathogen]* may underlie at least some cases of amaurosis. It is fascinating to see that modern histopathology indeed revealed IgG and complement deposits (*Ablagerungen*) in optic nerve and spinal cord lesions from patients with NMO [56–59], a feature not present in most cases of classic MS [60, 61]).

### ‘Spinal amaurosis’ in the medical literature of the first half of the 19th century

**Fig. 4:**
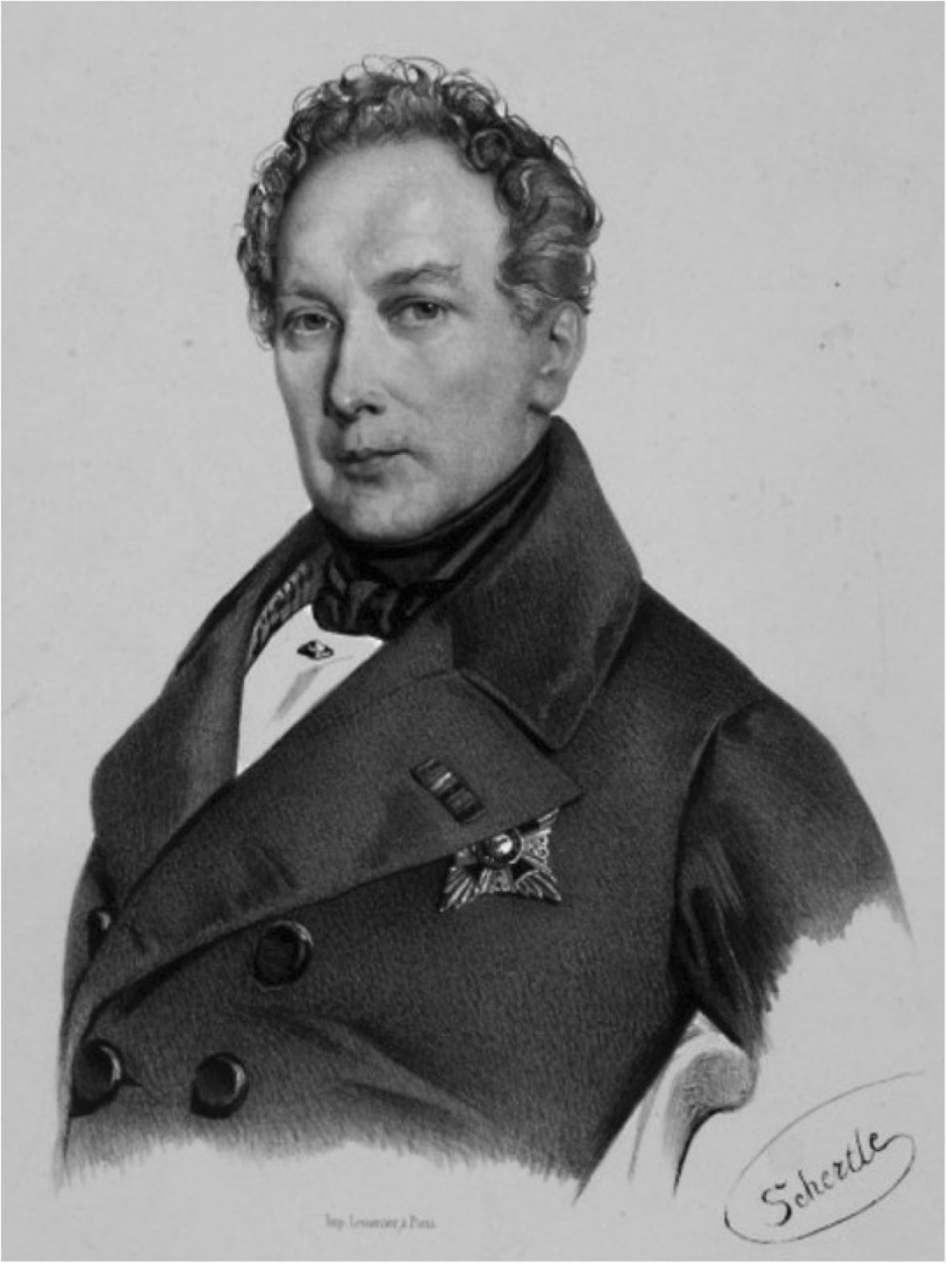
Maximilian Joseph (von) Chelius (1794–1876)

Sichel’s concept of ‘spinal amaurosis’ was soon further popularised by a number of translations of his treatise [62, 63] and by its discussion in Heimann Breßler’s *Die Krankheiten des Kopfes und der Sinnesorgane* (in three volumes, 1840) [64] and in Maximilian Joseph Chelius’s (1794–1876) *Handbuch der Augenheilkunde* (1843) [65]. Especially Chelius’ reputation – he had already published his highly influential *Handbuch der Chirurgie* (1822–1857; 8 editions and 11 translations) at that time and was one of the best-known surgeons in Europe (Fig. 4) – may have been helpful in gaining acceptance for Sichel’s concept, all the more since Chelius in his textbook distinguished Sichel’s and von Walter’s contribution to the understanding of amaurosis as follows: *„So wie Beer zuerst die genauere Symptomatologie der verschiedenen Formen des schwarzen Staares aufgestellt hat, so haben Sichel und v. Walther am meisten zur wissenschaftlichen Begründung der Lehre des schwarzen Staares beigetragen* “*[Just as Beer first established the exact symptomatology of amaurosis, Sichel and v. Walther contributed most to the scientific foundation of the nosology of amaurosis]*. Slightly modifying Sichel’s wording (“*spinale Amaurose*” in the German translation of Sichel’s *Traité*), Chelius used the composite term “*Spinal-Amaurosen*” *[spinal forms of amaurosis]*, which he differentiated from “*Cerebral-Amaurosen*” *[cerebral forms of amaurosis].* Among the symptoms that may indicate “*Spinal-Amaurose*”, he listed lumbar or dorsal pain, paraesthesia, numbness, paresis and/or spasticity of the extremities, girdle dysaesthesia, respiratory distress, cardiac arrhythmia, bladder and bowel disturbances and impotence.

Besides Chelius, also Karl Himly (1772–1837) in his widely read classic *Die Krankheiten und Missbildungen des menschlichen Auges und deren Heilung* (1843; posthumously edited and published by his son Ernst August Wilhelm Himly [1800–1881]) [66], which appeared in the same year as Chelius’ textbook, took up Sichel’s concept of ‘spinal amaurosis’. Importantly, Himly, like Carron du Villards before him, explicitly referred to myelitis as a potential cause of amaurosis: “*β) [Amaurosis durch] Überreizung des Rückenmarks. An Myelitis Leidende werden zuweilen amaurotisch: Carron du Villards sah die Blindheit mit dem Aufhören jener Entzündung verschwunden* “*[Amaurosis caused by irritation of the spinal cord. Patients suffering from myelitis occasionally become amaurotic: Carron du Villards recognised that the amaurosis vanished together with that (*i.e. *spinal) inflammation]*.

Sichel’s idea also found its way into the French literature. While Auguste Théodore Vidal (1803–1856) in his 1840 *Traité de pathologie externe et de médecine opératoire* [67] limited himself mainly to reproducing Sichel’s classification of amaurosis, which included spinal amaurosis as one category among many, later authors mocked Sichel’s (and other German authors’) indeed somewhat scholastic and, to some extent, speculative system of classifications of amauroses *(“l’imagination puissante mais aventureuse de l’Allemagne émet toute une classification d’amauroses, dont l’interminable nomenclature semble nécessiter des prodiges d’analyse et ne nécessite que des prodiges de mémoire” [the powerful yet adventerous German imagination emits a whole classification of amauroses, the endless nomenclature of which seems to require prodigies of analysis yet only requires prodigies of memory]* [68]) or tried to put the relevance of co-existing spinal diseases into perspective (*“Il ne faut pas s’exagérer l’importance de l’intervention de la moelle épinière sur la production des phénomènes amaurotiques” [The importance of the contribution of the spinal cord on the generation of amaurotic phenomena should not be exaggerated]* [22]). While Charles Deval in his *Traité de l’amaurose ou de la goutte-sereine* (1851) widely followed his teacher Sichel in his definition of spinal amaurosis (*“[d] es phénomènes amaurotiques viennent-ils s’ajouter aux symptômes de la myélite, du ramollissement de la moelle épinière, d’une altération quelconque de celle-ci ou de ses annexes” [amaurotic phenomena come along with the symptoms of myelitis, softening of the spinal cord, any alteration of the spinal cord or its annexes]* [22]), he also critically added for consideration that the validity of some aspects of Sichel’s concept were *“loin encore d’ètre démontrée” [far from being proven]*.

In England, the concept of spinal amaurosis was introduced by Edward Octavius Hocken, a young ophthalmologist, whose reports on patients with amaurosis and spinal cord disease we recently re-discovered [69]. Whether Hocken was familiar with Sichel’s works or whether he developed the concept independently in parallel with Sichel we cannot know with certainty. However, in his *Pathology and Treatment of Amaurosis*, published in *The Lancet* in 13 parts between March 1841 and April 1842 [70–73] and, in parallel, in *The London Medical Gazette* [74], Hocken referred solely to his own experience, which, as he was convinced, “*proved the occasional dependence of imperfection or loss of vision solely on spinal disease*” – a dependence that, as he pointed out, “*seems to have been overlooked by all previous ophthalmological writers*”.

### ‘Spinal amaurosis’ in the second half of the 19th century

The term ‘spinal amaurosis’ remained in use throughout the entire 19th century. However, it was not always used to refer to the association of acute amaurosis and myelitis. Already Sichel had adverted to debauchery (i.e. venereal diseases) and Chelius to tabes dorsalis as possible causes of spinal amaurosis. Von Walther, in his *Die Lehre vom schwarzen Staar und seine Heilart. Pathologie und Therapie der Amaurose* [75], even limited the symptoms of ‘spinal amaurosis’ to those of tabes dorsalis. This said, it should be taken into account that von Walther, a trained ophthalmologist, might well have intermingled cases of tabes dorsalis and acute myelitis as can be seen from his definition of the former disease: *“Die Symptome der Tabes dorsalis (...) Paralysis oder Paresis der untern Extremitäten; Incontinenz des Urines.”* *[the symptoms of tabes dorsalis (…) paralysis or paresis of the lower extremities; urinary incontinence]*. In the second half of the century, more and more authors – including such eminent ophthalmologists such as Carl Ferdinand Ritter von Arlt (1812-1887) [76], his pupil von Graefe [77, 78], and some of Graefe’s own pupils such as Richard Liebreich [1830-1917] [79]), as well as parts of the medical press [80] – would use the term preferentially or exclusively in that particular sense (referred to as ‘amaurosis tabidorum’ by some authors [64, 81–84]), while the association of amaurosis with myelitis suggested by Sichel, de Villards and Himly would be neglected. Eugène Follin (1823–1867) in his *Leçons sur l’application de l’ophtalmoscopie* (1859/1863), the earliest French work devoted entirely to the ophthalmoscope [85], Dixon (1855/1866), in his well-known ophthalmological textbook [86, 87], Richard Förster (1825-1902), in his chapter on neurological diseases in Gräfe und Saemisch’s *Handbuch der gesammten Augenheilkunde* (1876), the most comprehensive ophthalmological textbook of the time [88], and Ernst Pflüger (1846-1903), in his oft-cited 1878 review on optic neuritis in *Gräfes Archiv für Ophthalmologie* [89], did not even mention the concept of ‘spinal amaurosis’. Similarly, many of the most distinguished exponents of the newly emerging discipline of neurology were not aware of the association: Neither Moritz Heinrich Romberg (1795-1873) in his *Lehrbuch der Nervenkrankheiten des Menschen* (several editions between 1840 and 1857), which contains a widely read chapter on amaurosis, nor Karl Ewald Hasse (1810–1902; teacher of Robert Koch and Wilhelm Wundt) in his comprehensive chapter on myelitis in *Krankheiten des Nervenapparates* (1855), another reference textbook of the time, refer to the syndrome. Ernst von Leyden (1832-1910) had still pointed to the presence of amaurosis in spinal cord diseases other than tabes dorsalis in his 1863 monograph *Die graue Degeneration der hinteren Rückenmarksstränge* in a footnote: *“Es ist übrigens nicht unwahrscheinlich, dass sich die Atrophie des Opticus auch zu anderen Rückenmarkskrankheiten [als dem Tabes dorsalis] gesellen kann” [It is, by the way, not improbable that optic nerve atrophy may be associated with spinal diseases other than tabes dorsalis] *[90] but did not make any mention any more of that association in his renowned *Klinik der Rückenmarkskrankheiten*, which appeared in two volumes in 1874 and 1876 [91]. Missing out on Sichel’s, Carron du Villards’, and Pétrequin’s observations – as well as on the early reports by Edward Hocken (1820–1845) [69], Giovanni Battista Pescetto (1806–1884) [31], Christopher Mercer Durrant (1814–1901) [92], Jacob August Lockhart Clarke (1817–1880) [93], and Thomas Clifford Allbutt (1836–1925) [94] recently rediscovered by us –, Wilhelm Heinrich Erb (1840–1921), considered by many the founding father of German neurology, would declare in 1878 in his reference book on diseases of the spinal cord, “*von Störungen der Sehnerven ist bei der acuten Myelitis nichts bekannt*” *[nothing is known of disturbances of the optic nerves in patients with acute myelitis]* [95]. In 1880, on the occasion of his report on the first known German patient with NMO (published in parallel by Erb and Philipp Stephan, a Frankfurt ophthalmologist [96]), Erb would still claim that such an association had been widely unknown before [97]. Similarly, William Gowers (1845–1915), then the most eminent British neurologist, would state, in an address on *Eye symptoms in diseases of the spinal cord* delivered before the Ophthalmological Society, in 1883 [98, 99]: *“I have ( …*) *never [seen optic nerve damage] in ( …*) *myelitis”*. Also in Charcot’s lectures we were not able to find any reference to cases of spinal amaurosis other than tabetic amaurosis.

However, the association of amaurosis and spinal cord disorders other than tabes had never been *completely* forgotten. Christian Georg Theodor Ruete (1810–1867), both in the first (1845) and the second edition (1853/1854) of his *Lehrbuch der Ophthalmologie für Aerzte und Studierende* [100, 101], Desmarres (1810–1882), a former assistant to Sichel, in his *Traité théorique et pratique des maladies des yeux* (1847 and 1858; German translations in 1852 and 1868) [102] and Tetzer in his *Vorlesungen* (published posthumously in 1870; further editions in 1874 and 1887) [103] all mentioned tabes dorsalis as an *example* of ‘spinal amaurosis’, implying that other spinal causes existed: *“Es gibt eine Reihe von Krankheiten des Rückenmarks, welche mit Amaurose verbunden sind […] [A]m häufigsten […]* *Tabes dorsalis”* *[quite a few diseases of the spinal cord exist that are associated with amaurosis, the most frequent being tabes dorsalis]* [103]. Ruete explicitly pointed to the peculiar case of the Marquis de Causan, first reported by Antoine Portal (1742–1832) in 1804 and re-presented by John Abercrombie (1780–1844) in 1828, which is the first known account in the Western literature of visual loss in a patient with spinal cord inflammation but no brain pathology [104]; in that patient, no signs of tabes or locomotor ataxia were noted. Similarly, Carl Stellwag von Carion (1823–1904) in the 1861/1862 edition (as well as in later editions) of his highly successful *Lehrbuch der praktischen Augenheilkunde* [105] (at least four English editions would appear between 1868 and 1873; also translated into Italian and Hungarian) reminded of *“Amaurosis spinalis”*. He stressed that not only tabes dorsalis but various disorders of the spinal cord – including spinal cord inflammation – may be associated with amaurosis: “*Jedenfalls ist in Rechnung zu stellen, dass entzündliche Affection [des Sehnerven] (...) und dieses oder jenes Rückenmarksstranges fast gleichzeitig und ganz unabhängig von einander gar nicht selten beobachtet wird.” [In any case, we must keep in mind that almost simultaneous and independent inflammatory affection [of the optic nerve] (…*) *and of this or that spinal nerve fibre bundle is not infrequent]* [105], though he conceded that the exact relationship of the two affections remained a riddle: *“Der Zusammenhang des Spinalleidens mit dem schwarzen Staare ist bisher noch ganz dunkel geblieben” [The relationship of spinal disease with amaurosis is still totally obscure]* [105]. Eugène Bouchut (1818–1891), whose textbook on ophthalmoscopy (entitled *Du diagnostic des maladies du système nerveux par l’ophthalmoloscopie* 1866) appeared 5 years before Allbutt’s [106] and 13 years before Gower’s [107], was convinced that the introduction of the ophthalmoscope would make it possible to diagnose diseases not only of the brain but also of the myelon: *“Si la découverte de cet instrument a été l’origine de progrès importants pour l’étude des maladies de l’oeil, sachons qu’il peut être la source de progrès non moins précieux dans le diagnostic des maladies cérébro-spinales en nous donnant le moyen de découvrir au travers de l’oeil les altérations qui se produisent dans les différentes parties du cerveau et de la moelle*” *[If the discovery of this instrument (*i.e. *the ophthalmoscope) was the origin of significant progress in the study of diseases of the eye, it can be the source of no less valuable progress in the diagnosis of cerebro-spinal diseases by giving us a means to discover through the eye alterations that are produced in different parts of the brain and spinal cord]* [108]. He explicitly distinguished amaurosis associated with locomotor ataxia (i.e. tabes dorsalis) from amaurosis in patients with ordinary myelitis. In Édouard Meyer’s (1838–1902) influential textbook *Traite pratique des maladies des yeux* (four French editions between 1873 to 1895), which is based on his 1863 lectures at the *Ecole pratique* of the University of Paris and which appeared in German, English, Russian, Polish, and Spanish translations throughout the 1870s and 1880s, we read: *“L’amaurose spinale se rencontre surtout dans les cas de dégénérescence grise des cordons postérieurs (tabès dorsalis), plus rarement dans la myélite des cordons latéraux*” *[Spinal amaurosis occurs mainly in cases of grey degeneration of the posterior cords (tabes dorsalis), more rarely in the myelitis of the lateral cords]* [109]. Meyer also explicitly mentioned paraplegia, a manifestation not typically associated with tabes dorsalis (which is primarily a slowly progressive degenerative disease of sensory nerves and ganglia in the dorsal columns and sensory roots [110]), among known accompaniments of amaurosis. Meyer, a pupil of von Graefe, carried on Sichel’s work in Paris and purchased his clinic [111]. Theodor Leber, nowadays mostly remembered as the eponym of Leber’s hereditary optic neuropathy, dedicated a whole chapter to the topic, entitled *Spinalamaurose*, in Graefe und Saemisch’s *Handbuch der Gesammten Augenheilkunde* (1877). While he mainly dealt with the subject of tabes dorsalis in that chapter, he also pointed to the fact that other spinal disorders may rarely cause amaurosis *(“doch ist Ihr Vorkommen auch bei anderen Rückenmarksleiden nicht selten”*). However, of all the previously published cases, he was only aware of Allbutt’s cases, incorrectly stating that *„über Sehnervenleiden bei acuten Rückenmarksaffectionen ist [sonst] nur wenig bekannt* “*[about optic nerve diseases in spinal cord affection (otherwise) little is known]* [112]).

The rare association of amaurosis with spinal cord disorders was also recognised by English-speaking authors: The 1854 edition (but not earlier editions) of William Mackenzie’s (1791–1868) *A practical treatise on the diseases of the eye* [113], one of the leading British textbooks on ophthalmology of the period, listed “diseases of the spinal cord” among possible “complications of amaurosis”, thus suggesting a causal, pathogenetic relationship between the two events. (When studying Mackenzie’s work, the reader should carefully note that the term ‘paralytic amaurosis’ used elsewhere in that book refers – following Beer’s nomenclature – to cases of amaurosis associated with paralysis of eye muscles but not to such of amaurosis and paralysis of the limbs.) The term ‘spinal amaurosis’ was also kept alive by von Graefe’s former assistant John Soelberg Wells (1824–1879) in his *Treatise on Diseases of the Eye* (1869 [114]; 3rd edn. 1873; 4th American edn. 1883; translated into French and German), which according to *Plarr’s Lives of the Fellows of the Royal College of Surgeons of England* embodied *“the best teaching and practice of Continental and British practice”* of its decade and *“the standard textbook on the subject”*. Wells maintained the view that, *“The affection of the optic nerve in diseases of the spine is probably due to a lesion of the great sympathetic through its communication with the anterior roots of the spinal nerves”.* Similarly, Boston-based Henry Angell (1829–1911) would refer to “*spinal disorders*” in general rather than to tabes in particular in his 1870 *Treatise on diseases of the eye* [115], which went through seven editions between 1870 and 1891, and John Phillips, one of the instigators of the use of the ophthalmoscope in the USA [116], explicitly referred to amaurosis “complicated with (…) paraplegia” and with inflammation of the spinal cord in his 1869 textbook *Ophthalmic surgery and treatment* [117]. Notably, Phillips also mentioned “morbid changes about the medulla oblongata” as a “precursor of spinal amaurosis”. Today, we know that NMO is rather frequently associated with brainstem lesions and that lesions of the medulla oblongata, typically causing intractable vomiting and/or hiccups, indeed often herald the onset of NMO [1, 10, 118]. Finally, Allbutt would take up the term in his textbook *On the use of the ophthalmoscope in diseases of the nervous system and of the kidneys* (1871) [106], in which, tucked away in the appendix, a more detailed description can be found of the patient with ON and acute myelitis that he had briefly mentioned in his famous lecture on the ophthalmoscopic signs of spinal disease, published the year before in the *Lancet* [94, 119]. Had Allbutt used the term ‘spinal amaurosis’ in the *Lancet* article, which most likely had a much broader readership than his book and which has been so often (falsely) cited as the first mention of a patient with NMO, the term might not have fallen into oblivion. Finally, Henry Swanzy (1843–1913) in his *Handbook of the diseases of their eye and their treatment* (1892/1915) also refers to *“spinal amaurosis”* [120, 121]. Swanzy also counted insular sclerosis (i.e. MS) among its possible causes.

Numerous cases of probable NMO were published during the 1880s and 1890s (e.g., [97, 122–134]), and in 1893 and 1896, respectively, two German reviews appeared (both entitled *Über das Zusammenvorkommen von Neuritis optica und Myelitis acuta [On the coincidence of optic neuritis and acute myelitis]*), one authored by the Dresden-based ophthalmologist Fritz Schanz [130, 135] and one by Karl Katz, the aforementioned doctoral student to Leber in Heidelberg [136]. However, it was mainly Devic’s report of a case of “*myélite aiguë dorso-lombaire avec névrite optique*”, communicated on the occasion of the Congrès Français de Médecine in Lyon in 1894 [133, 137, 138], and the thesis of his student Fernand Gault published shortly thereafter [44], a review of the previous literature (based on Schanz’s review, as acknowledged by Devic in [137]), which created new and sustained interest among neurologists and ophthalmologists in this rare syndrome at the end of the 19th century.

By the end of the century, the concept of NMO was widely accepted. Von Leyden atoned for his former omission in his monumental, 970-page standard textbook *Die Erkrankungen des Rückenmarkes und der Medulla oblongata* (1897; together with Johannes Goldschneider [1858–1935]) [139], in which he acknowledged the co-occurrence of myelitis and ON. As a limitation, however, Leyden referenced Erb’s 1879 case [97] as the first description (*“zuerst von Erb [beobachtet]” [first observed by Erb]*), overlooking – just like Erb before him – all earlier cases, including those reported by Sichel, Carron du Villards, Pétrequin, Hocken [69], Pescetto [31], Durrant [92], Clarke [93] and Allbutt [94]. During the following twentieth century, NMO would often be mistaken as a variant of MS. It is therefore of note that von Leyden pointed to the fact that spinal amaurosis is distinct from MS in some cases according to autopsy findings: *„In einigen Fällen von Myelitis, auch solchen, bei welchen durch die Autopsie die Diagnose gesichert worden ist, ist Neuritis optica beobachtet worden (...) Es scheint sich vorzugsweise, aber doch nicht ausschließlich, um die disseminierte Form der Myelitis zu handeln* “*[In some cases of myelitis, including cases in which the diagnosis was confirmed by autopsy, neuritis optica has been observed (…*) *It seems these cases represent preferentially but not exclusively the disseminated form of myelitis]* [139]. Etiologically, von Leyden considered those cases to be of mostly postinfectious origin. In the second, revised edition in two volumes (1902/1904), he would repeatedly refer to the coincidence of acute myelitis and ON (citing a number of then recent clinical and pathological studies [125, 129, 134, 140, 141] on NMO), which he recognised as an important differential diagnosis of neurosyphilis.

### Sichel’s primacy in coining the term ‘spinal amaurosis’

Considering that there is rarely such thing as a new idea, we performed a careful analysis of much of the early 19th century French, German, English and Italian ophthalmological literature that preceded Sichel’s report, including, among others, the works of Trnka von Kržowitz (1781/1791) [142, 143], Beer (1791 [144], 1792 [145, 146] and 1813/1817 [147, 148]), Richter (1795 [149]), Himly (1801 [150], 1804 [151], 1806 [152] and 1830 [153]), Scarpa (1801 [23] and 1803), Wenzel (1808 [154]), Kieser (1811 [155]), Wardrope (1808 [156] and 1818 [157]), Demours (1818 [158]), Guillie (1818 [159]), Langenbeck (1815/1818 [160]), Vetch (1820 [161]), Travers (1820 [162] and 1825 [163]), Stratford (1828 [164]), Lawrence (1830 [165] and 1833 [166]), Fischer (1832 [167]), Beck (1832 [168]), Andreae (1834 [169]), Bessières (1838 [170]), Middlemore (1835 [171]), Weiss (1837 [81]), Langenbeck (1815/1818 [160]) and Rognetta (1839 [172]), to verify Sichel’s primacy in coining the term ‘spinal amaurosis’. This extensive search revealed no earlier instance of the use of the term.

Of particular note, the term was also unknown to Carl Heinrich Weller (1794–1854) in his *Die Krankheiten des menschlichen Auges* (1819 [173]; 1826 [174], English translation 1821 [175, 176]; French translations 1828 [177] and 1832 [178]), on the third edition of which Sichel’s *Traité de l’óphthalmie, la cataracte et l’amaurose* was explicitly based, as openly acknowledged by Sichel (“*pour server de supplement au traité de Mr. Weller*”).

## Amaurosis and spinal cord disease before Sichel

While the term ‘spinal amaurosis’ thus seems indeed to have been introduced by Sichel, the fact that optic nerve and spinal cord disease may occasionally co-exist had not completely escaped the attention of ophthalmologists and pathologists:

- The year before Sichel’s report appeared, Bernhard von Langenbeck (1810–1887), who would later become founder – together with Billroth, Volkmann, Trendelenburg and others – and almost life-long president of the German Society of Surgery, had published his Latin-language dissertation *De retina observationes anatomico-pathologicae* (1836), in which he pointed to cases in which softening of the spinal cord (myelomalacia) – a term applied by some 19th century authors to refer to myelitis [91] – was associated with softening of the optic nerve (ophthalmoneuromalacia) or the retina (amphiblestrodomalacia). Of note, Sichel was explicitly aware of Langenbeck’s observation: *“M. Langenbeck jeune pense qu‘un grand nombre des ces cas d’amauroses peuvent s’expliquer par la co-existence du ramollissement de la rétine avec le remollissement de la moelle épinière, assertion dont l’expérience seule peut confirmer la valeur” [Langenbeck jun. thinks that a large number of these cases of amaurosis can be explained by the co-existence of the softening of the retina with the softening of the spinal cord, an assertion the value of which experience alone can confirm]* [8].
- Previously, August Andreae (1794–1867) in his *Grundriss der allgemeinen Augenheilkunde* (1834) [169], which antedates Sichel’s *Traité de l’ophthalmie* by 3 years, had already referred to “*Lähmung des ( …*) *Rückenmarks*” *[paralysis of the spinal cord]* as a possible cause of amaurosis. Unfortunately, however, Andreae did not go into more detail regarding the nature and causes of that ‘paralysis’. (The use of the term ‘paralysis’ in regard to nervous system structures such as the spinal cord or the optic nerve rather than to voluntary muscles was not uncommon but had already been subject to criticism by contemporaries of Andreae; see, for example, the 1825 edition of Travers’s *A synopsis of the diseases of the eye, and their treatment*, the earliest systematic treatise in English on diseases of the eye: *“To apply the term paralysis to a nervous tissue, is, to say the least of it, a misnomer. A muscle may be paralysed from pressure or injury of the nerve which supplies it, or the part of the brain or spinal marrow whence that nerve is derived; but there is certainly nothing in the nerve itself sufficiently analogous to muscular structure or function to apply the term ‘paralysis’ to both”*).
- Karl Himly, Christoph Wilhelm Hufeland’s (1762–1836) successor in Jena, in an article entitled *Bemerkungen über die Hauptarten der Amblyopie und Amaurose*, published in 1804 in the second volume of his *Ophthalmologische Bibliothek*, had pointed to cases of amaurosis in patients with spasticity of the voluntary muscles, especially those of the lower extremities. However, he, too, neither used the term ‘spinal amaurosis’ or made any reference to inflammation of the spinal cord [151]. Himly’s remarks were adopted almost verbatim in later medical textbooks of the period (e.g., in Schmalz’s *Versuch einer medizinisch-chirurgischen Diagnostik in Tabellen* 1816/1825/1830 [179]).
- Charles-Prosper Ollivier d’Angers (1796–1845), author of *De la moelle épinière et de ses maladies* (1824; a second – doubled in size and in two volumes – and a third edition would appear in 1827 and 1837, respectively), a monumental, pioneering and then highly influential work, which included the most comprehensive collection of case studies in its field yet assembled, and which would be also translated into German (1824), Italian (1835–1839) and English (1843), agreed that myelitis may be accompanied by visual loss but maintained, *“Ces accidens sonst bien évidemment la suite de l’irritation portée vers le cerveau ou ses membranes” [These coincidences are of course the result of (additional) irritation to the brain or its membranes]* (cited from the first edition) [180].
- Antoine Portal (1742–1832), tutor of the young Louis XVI, first physician to Louis XVIII and to Charles X, and founding and lifelong president of the Académie Nationale de Médecine, in the fourth volume of his *Cours d’Anatomie Médicale, ou Élémens de l’Anatomie de l’Homme* (1803–1804) recalled a case (recently rediscovered by us [104]) of bilateral blindness, tetraparesis, respiratory distress and dysphagia. At the opening of the body, the spinal cord contained in the cervical vertebrae was found to be very hardened, of a cartilaginous consistency, and the surrounding membranes were noted to be red, as if inflamed. By contrast, the brain was in a completely healthy state (as were all other parts of the body). No signs or symptoms of syphilis were noted. The case was made known to a broad audience by virtue of often being cited and discussed in British, North American and French books and articles by John Abercrombie (1780–1844) [181, 182], Ollivier [183, 184], Edward Meryon (1809–1880) [185] and others [104].
- Finally, Beer, Sichel’s teacher, who is considered by many the founder (together with von Walther) of modern ophthalmology in Germany, in the second volume of his *Lehre von den Augenkrankheiten* (1817; English translation 1823) [147, 148] had pointed to concussion of the spinal cord as a rare suspected cause of amaurosis (*“durch Erschütterungen des Rückenmarks, durch den Fall von einer bedeutenden Höhe mit der ganzen Last des Körpers auf die Fersen” [due to concussion of the spinal cord, by falling from substantial height with the full weight of the body on one’s heels]*). Beer’s concept of what could be named ‘post-traumatic spinal amaurosis’ was made known to a broader audience by its being cited – despite early criticism (Lawrence 1833 [166]) – in numerous textbooks as well as encyclopaedias (see, for example, Schmalz 1816 [179], Weller 1819 [173], Vetch 1820 [161], Cooper 1822 [186, 187], Frick 1823 [188], Schmalz 1825 [189], Frick and Wellbank 1826 [190], Rosas 1830 [191], Schmalz 1830 [192] and Cooper 1836 [193]) and thus may well have sensitised Sichel to the rare coincidence of spinal cord and optic nerve affections. However, just like Himly, Beer never used the term ‘spinal amaurosis’ and did not investigate spinal inflammation as a possible cause of amaurosis. The topic continued to generate long-standing interest among ophthalmologists (e.g., Desmarres 1847 [102], Lawrence 1854 [166, 194], Cooper 1854 [195], Soelberg Wells 1869 [114], Wharton Jones 1869 [196], Allbutt 1870 [106, 119], Mooren [197, 198], Oglesby 1874 [199], Thorowgood 1875 [200, 201], Erichsen 1875 [202], Duplay and Follin 1875 [203], Pflüger 1878 [89], Meyer 1879 [204], 1883, 1887 and 1895, Clarce 1880/1881 [205], Firth 1886 [206], and Taylor 1901 [207]) and should become a matter of heated debate in the 1870s following the introduction of the ophthalmoscope and, in particular, the publication of Wharton-Jones’ *Failure of sight from railway and other injuries of the spine and head* in 1869, and would be harshly criticised by Gowers [206]. The discussion about the exact mechanism by which spinal cord injury could possibly cause optic nerve damage (nutritional damage to the optic nerve resulting from damage to the spinal origins of the sympathetic nerves vs. ascending meningeal ‘irritation’ or meningitis) would later on also determine the discussions on pathomechanisms underlying the simultaneous or successive occurrence of myelitis and ON in NMO for a long time.
- In addition, we came across a number of case reports compatible with (and in at least one case highly suggestive of) NMO, all of which predate Sichel’s 1837 report by years or decades. While these reports (to be presented in the following sections), some of them authored by eminent figures of early 19th century medicine in Great Britain, do not challenge Sichel’s primacy in coining the term ‘spinal amaurosis’, they further demonstrate that the syndrome itself had already been recognised by ophthalmologists and pathologists before Sichel.

### Matthew Baillie (1820, 1822 and 1824): an early NMO case series?

**Fig. 5:**
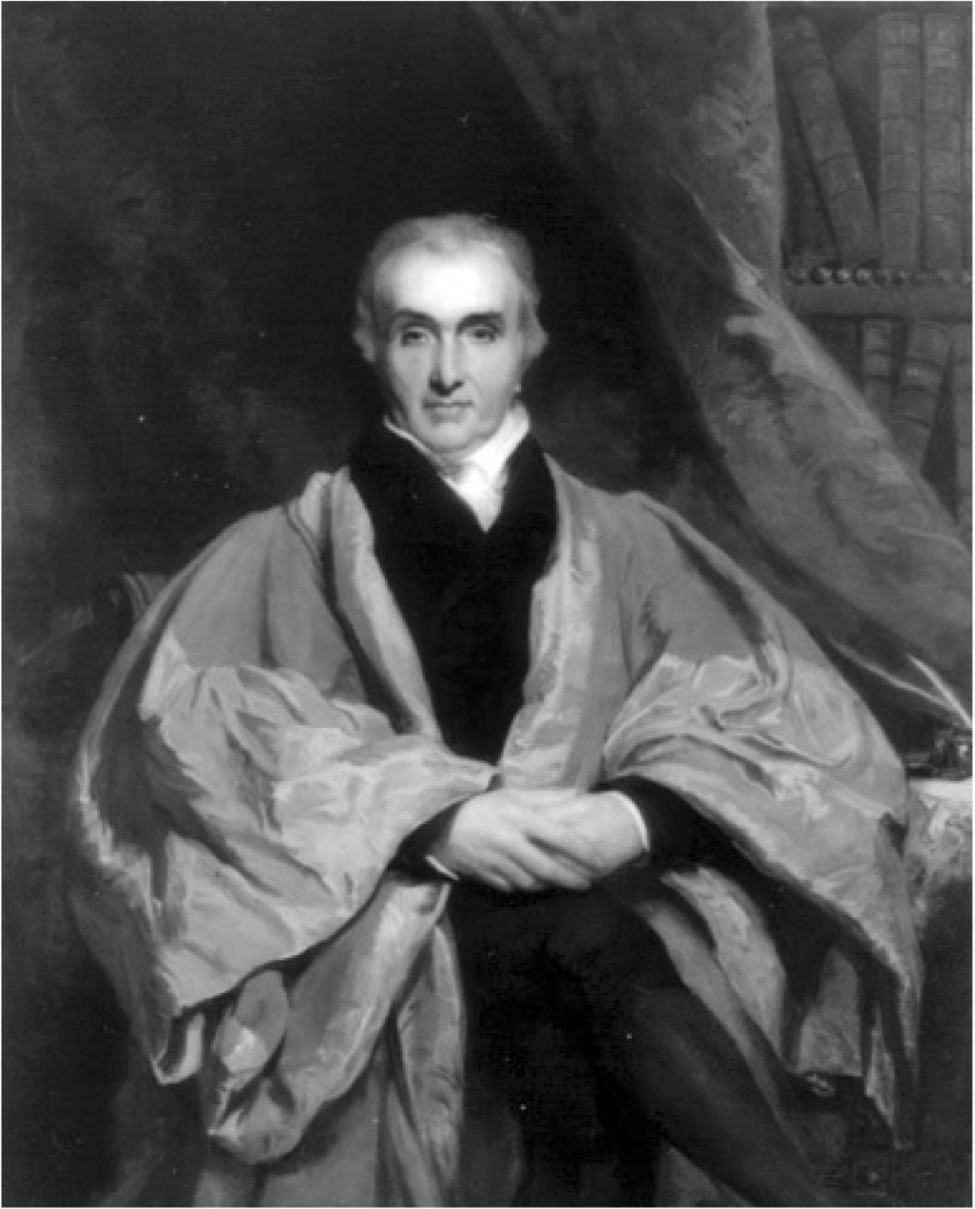
Matthew Baillie (1761–1823)

First, we would like to draw the reader’s attention to a very early case series of patients with ‘gutta serena’ (a historic term for blindness) and para- or tetraplegia collected by Matthew Baillie (1761–1823) (Fig. 5), a Scottish-born British physician, anatomist and pathologist, shortly before his death in 1823. Baillie’s notes were made known to the medical public only posthumously in 1826.

Baillie, who had been made Fellow of the Royal Society (FRS) in 1790, was nephew and pupil to the renowned anatomists John Hunter, FRS (1728–1793), and William Hunter, FRS (1718–1783), was educated at Oxford, and is nowadays mostly known for his celebrated *Morbid anatomy of some of the most important parts of the human body* (1793) [208, 209], the first systematic textbook on pathology in English and the first to make pathology a subject in itself (see ref. [210] for a biographic sketch). He is also considered to have given the first description of a patient with *situs inversus viscerum*. Finally, readers interested in the history of medicine may be familiar with Baillie as the last owner of the famous gold-headed cane originally possessed by no less figure than Oxford’s great benefactor John Radcliffe (1652–1714) [211].

In 1820, in a paper entitled *Some observations on paraplegia in adults* and published in the sixth and last volume of the *Medical Transactions* of the London College of Physicians [212], Baillie proposed that cerebral lesions could cause paraplegia (*“[a condition] in which the lower half of the body is more or less impaired in its nervous power”*). As an example for brain involvement in patients with paraplegia, he referred to patients with concomitant optic nerve damage (anatomically, the optic nerve is part of the brain): *“Sometimes the sight of one eye is almost entirely lost, and its pupil appears dilated as in gutta serena”* [212].

While the hypothesis of cerebral disease being capable of causing paraparesis was considered acceptable by many of Baillie’s contemporaries, his opinion that *“if the spine has not suffered outward violence, paraplegia* most commonly (our emphasis) *depends on a disease of the brain itself”* was met with reservation by some and openly rejected by others (see, for example, reference [213]: *“[I]t is not a little remarkable that one whose opportunities and whose powers of observation were so good, was led to adopt such views. Can it be accounted for by supposing that, in those days, the spinal cavity was even less opened after death than at the present time?”*), all the more as Baillie gave results from only one dissection – a case of paraplegia associated with extensive effusion in the ventricles and membranes – in support of his theory. He later (in a brief paper dated June 1822 and posthumously published in an edition of his works arranged by James Wardrope in 1825 [214]) added three more cases, one caused by “*tumours in the brain*”, one in which “*many of the arteries of the brain were found ossified*”, and another in which “*a large quantity of water*” was found in the ventricles of the brain (but also “*some in the theca vertebralis*”).

However, Baillie obviously felt that these cases might not suffice to justify his far-reaching claim and continued to collect further cases, which he briefly described in a short letter he marked *Short memoranda of cases of paraplegia* on the back and entrusted to a colleague, a Dr. Gregory (probably George Gregory [1790–1853], the grandson of William Cullen’s [1710–1790] successor at Edinburgh, James Gregory [1753–1821], who had studied anatomy at the Windmill Street School under the tutelage of Baillie; Baillie and Gregory’s father were friends from their early lives at Balliol College, Oxford [215, 216]), to be produced should his opinions ever be particularly controversial [217]. All of those patients had developed amaurosis, which Baillie curiously took as sufficient proof that paraplegia was caused by cerebral disease as well. In 1826, these memoranda were read on the occasion of a meeting of the Westminster Medical Society (later printed verbatim – though now entitled *Facts relative to the paraplegia (From a posthumous MS. of the late Dr. Baillie*) [217]). In these memoranda, which sparked a lively discussion – including fair and objective contributions [217–221] as well as vituperative personal attacks [222] – Baillie briefly mentioned a series of five cases of amaurosis or impaired vision and para- or tetraparesis:

*1. “Another gentleman, in paraplegia, has gutta serena of his left eye; had great weakness in his arms, with indistinctive feeling, so that he said he could not distinguish, by the touch, shillings and sixpences from each other.”*

*2. “A lady, in paraplegia, had impaired vision, severe headache, and weakness in her arms and hands.”*

*3. “A clergymen had gutta serena of one eye along with paraplegia.”*

*4. “A gentleman had a temporary gutta serena, and an occasional dropping of one eyelid, with paraplegia.”*

*5. “A nobleman had the vision of both eyes very much impaired in paraplegia from* (sic!) *gutta serena, but his affection at length a good deal subsided.”*

While it can retrospectively not be proven that these patients had indeed AQP4-IgG- or MOG-IgG-related NMO, Baillie’s report again demonstrate that the peculiar coexistence of optic and spinal symptoms that characterises NMO, i.e. the isolated affection of two sites not apparently connected in terms of anatomy or function, attracted the attention of physicians much earlier than previously thought. With the exception of headache in one and occasional dropping of an eyelid in another patient, there was no indication for brain disease other than possible optic nerve involvement, which – though the absence of brain symptoms does not per se preclude early MS – renders a diagnosis of MS less likely.

As a limitation, it is unclear whether symptoms developed acutely – as typically seen in NMO – in Baillie’s patients. In consequence, tabes dorsalis cannot be completely ruled out. Some of the patients included by Devic and Gault in their seminal study of early NMO cases [44] did indeed have a history of syphilis, a disease that was highly prevalent throughout the 18th and 19th centuries. However, no such history was related by Baillie, nor did he report typical clinical symptoms of tabes dorsalis such as lancinating pain or paralytic dementia. Moreover, tabes dorsalis primarily affects the posterior columns; accordingly, paraplegia is not a typical manifestation. Finally, symptoms were transient in case 4 und substantial recovery was noted in case 5, in contrast to the chronic progression typically seen in neurosyphilis.

Regarding treatment, Baillie recommended, among other ‘remedies’ of the time, electrotherapy: *“In one case I found that a good deal of benefit was derived from electric sparks being drawn from the lower limbs”.* Electric treatment of patients with NMO would still be tried in the late 19th century [123]. To the best of our knowledge, Baillie’s report has never been cited again.

### Bilateral amaurosis following a violent attack of ‘pleuritis dorsalis’: James Ware (1795)

To assess whether Baillie and Portal were really the original ‘giants’ on the shoulders of which later generations stood (or sat, as the windows of the Southern transept of Chartres cathedral suggest [223]), we undertook to expand our search to the most important medical textbooks of the second half of the 18th century. However, any study of the history of NMO is complicated by the fact that modern ophthalmological and neurological terminology deviates from that used in the 18th and early 19th century.

Blindness was not always referred to by the name of ‘amaurosis’ at that time but also by a plethora of other terms, including ‘gutta serena’, ‘goutte-sereine’, ‘suffusio nigra’, ‘schwarzer Star’ or ‘amblyopia’ (weakness of sight).

Similarly, the term ‘myelitis’ was unknown until the early 19th century, when it was introduced to medical terminology by Johann Christian Friedrich Harleß as a substitute for the term ‘r(h)achialgitis*’* (in a footnote to a German translation of Brera’s *Della rachialgite, cenni patologici*, published in the *Jahrbücher der teutschen Medicin und Chirurgie, mit Zugabe des Neuesten und Besten aus der ausländischen medicinischen Literatur*, edited by Johann Christian Friedrich Harleß and published by Johann Schrag, Nuremberg, 1813, Vol. II, p. 244) and first needed to gain acceptance before it finally prevailed. In 1845 August Hennemann, in his *Die differentielle medizinische Diagnostik* would still list the following synonyms and related terms: *“Entzündung des Rückenmarks, Hyperämie des Rückenmarks, Plethora spinalis, Meningitis spinalis, Notaeomyelitis, Rhachiomyelitis (Funk), zum Theil die Pleuritis dorsalis und Angina vertebralis der Alten, Spinitis (Niel), Rhachialgitis (Brera), Spinodorsitis (Schmalz), Notiacomyelitis, Myelomeningitis, Perimyelitis)”* [224].

Of the latter terms – most of which are little known today – particular importance attaches to ‘notaeomyelitis’ and ‘pleuritis dorsalis’. Under the title of ‘notaeomyelitis’ (or, more precisely, its Italian equivalent ‘noteomielite’) Pescetto’s report – one of the earliest detailed reports on a patient with NMO – was published [225]. As pointed out by us in Part 1 of this article series [5], the term ‘notaeomyelitis’ was originally introduced by the Austrian physician Johann Valentin Hildenbrand (1763–1818), the eponym for Hildenbrand’s disease (i.e. typhus), in his textbook *Institutiones practico-medicae* (Vol. III [published posthumously in 1822], p.97ff) to distinguish the inflammation of the medulla spinalis or d o r s a l i s (termed μυελος ν ω τ ι α ι ο ς by Hippocrates) from that of other types of marrow such as the bone marrow (osteo-myelitis) or the cerebral white matter (encephalo-myelitis). The use of that rather unusual term may well have contributed to the fact that Pescetto’s early account of NMO went unrecognized for the next 150 years. Of special note, Hildenbrand was also the first to use the term neuromyelitis (or more precisely, nevromyelitis). However, by that term Hildenbrand was referring not to the co-occurrence of ON and myelitis, but to inflammation of the pulpa nervorum as opposed to that of the vagina nervorum (nevrilemmatitis).

‘Pleuritis dorsalis’, on the other hand, is a term that can be traced back to ancient times (see, for example, Maletius *De myelitide* 1837: *„Nominavimus morbum, de quo tres hoc ensus observatos descripsimus, m y e l i t i d e m. Varii medici varium huic morbo nomen dederunt. Nonnulli putant, eundem esse morbum, quem Hippocrates pleuritidem dorsalem nominavit”* [226]; and Georg Ferdinand Friedrich *De myelitide* 1825: *“Hippocrates […] morbum huncce pleuritides dorsalis nomine [descripsit]”* [227]). More specifically, the term could indicate dorsal myelitis as opposed to cervical myelitis (‘angina vertebralis’ [sic!]; see also Charles-Prosper Ollivier d’Angers [1796–1845] in *De la moelle épinière et de ses maladies* 1827: *“Elle peut simuler une angine”*) or lumbar myelitis (‘rachialigitis lumbalis’, or simply ‘lumbago’): *“Hippocrates modo anginae vertebralis, modo pleuritides dorsalis, modo lumbaginis usus est, prout vel haec vel illa medullae spinalis pars prae ceteris inflammatione laboraret. (…) Quodsi inflammatio praecipue partem cervicalem medullae offendit, symptoma cum iis anginae vertebralis Hippocratis conspirant, quiapropter Brera rachialgitidis vertebralis, Harles myelitides cervicalis nomen ei indiderunt. (…) Sin pars medullae dorsalis sedem inflammationis continet, symptoma illius affectionis apparent, quam Hippocrates pleuritidem dorsalem, Brera rachialgitidem dorsalem, Harles myelitidem dorsalem nuncupat. (…) Si denique pars lumbaris medullae inflammatur, Hippocrati lumbago, Brerae rachialigitis lumbalis, Harlesio myelitis lumbalis denominatur …”* [227]). That term, ‘pleuritis dorsalis’, was never completely forgotten (see, for example, Ballonius *Opera omnia medica in quatuor tomos divisa* 1634-1636; John Allen *Synopsis universæ medicinæ practicæ* 1719). Harleß, in the above-cited work, defined it as *“Rückenmarksentzündung” [inflammation of the spinal cord]* in 1813 and stated, *“Ueber diese Erkrankung finden sich einige nicht unbedeutende Andeutungen bei verschiedenen neuern Schriftstellern unter dem Namen der pleuritis dorsalis*” *[a number of not insignificant clues about this disease can be found in the works of various more recent authors under the name of pleuritis dorsalis]*. Georg Friedrich Most’s (1794–1845) *Encyklopädie der gesammten medicinischen und chirurgischen Praxis* from 1834 still covered ‘pleuritis dorsalis’, also defining it as ‘inflammatio medullae spinalis’ [84].

**Fig. 6:**
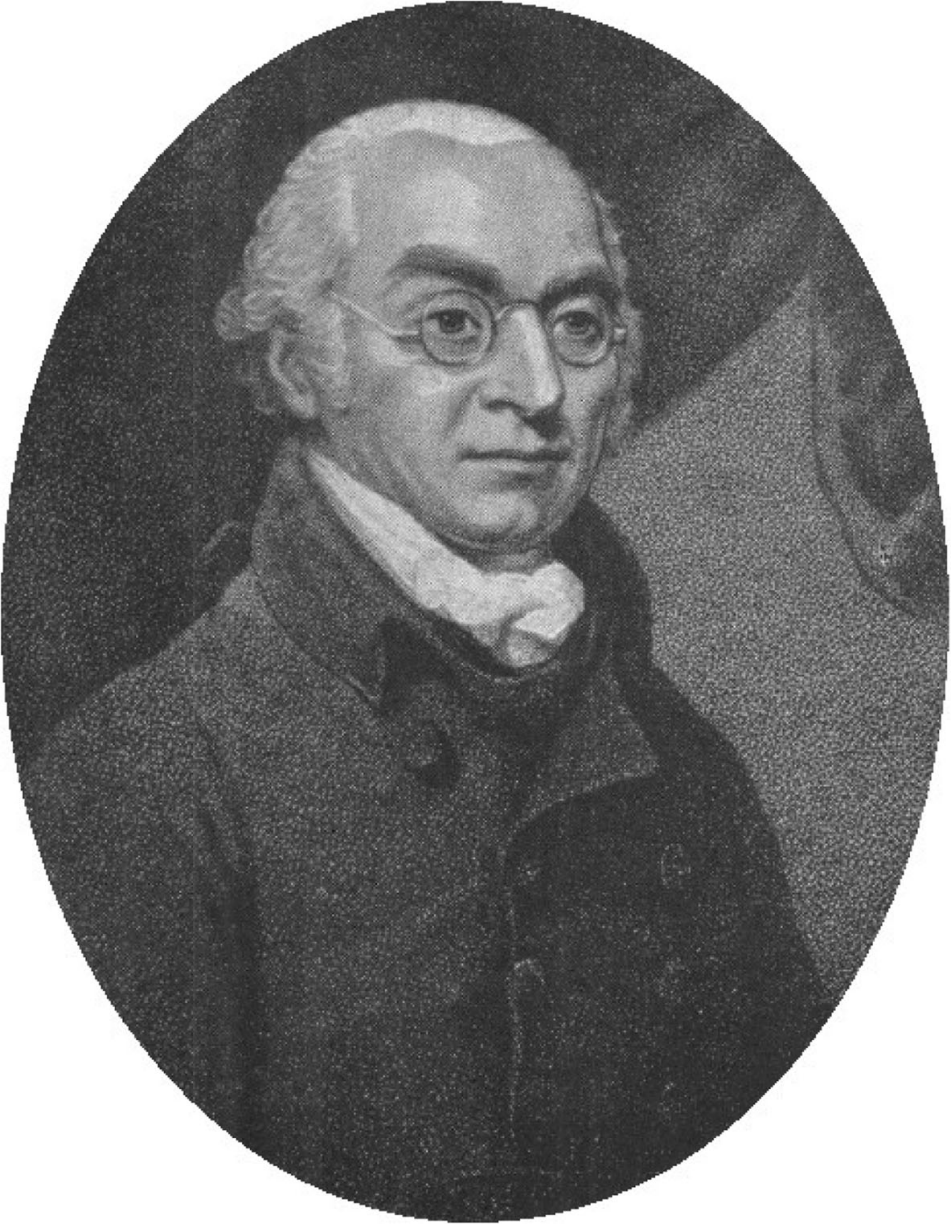
James Ware (1756–1815)

We believe it was in that sense that the term was applied also by James Ware (1756–1815) (Fig. 6) in his *Enquiry into the causes which have most commonly prevented success in the operation of extracting the cataract; with an account of the means by which they may either be avoided or rectified; to which are added observations on the dissipation of the cataract, and on the cure of the gutta serena* [228]), which first appeared in print in 1795, i.e. 18 years before Harleß would propose substituting ‘pleuritis dorsalis’ by the new term ‘myelitis’. In that treatise, Ware describes the case of a 45-year-old woman who experienced an attack of a *“violent pleuritic disorder”* which reduced her strength so much that she *“became even unable to turn herself in bed without assistance”*. One month after onset, she suffered from *“violent pain in the left eye”* and headache, which was soon followed by considerable loss in vision in that eye, which within 5 days became completely amaurotic. Three weeks later, she developed similar pain in the right eye, again followed by a decline in vision leading to almost complete blindness after 2 to 3 weeks and complete blindness after 1 month. At that time the violent eye pain ceased. Upon examination there were no signs of inflammation of the external eye structures, but the pupils of both eyes were found to be dilated with no apparent light reaction. The patient was removed into the country site, where she recovered her strength but not her sight. Treatments included vitriolic aether (i.e., diethyl ether), applied several times per day to both eyes, Peruvian bark, and other ‘remedies’. Of particular note, however, she was in addition treated by electrotherapy: *“a strong stream of the electric fluid was to have been applied to the eyes”* and continued for ten to fifteen minutes. While Ware was confident that this treatment had been *“not a little serviceable on every trial”*, it had to be ceased due to a little accident: *“the person employed in the business of the electricity, being unacquainted with the mode of applying the stream”, * accidentally substituted for it *“electric shocks (…) through the head”*. In consequence, the patient was *“electrified”* only three times, and *“in this way”*. Within 3 months time she recovered enough vision to enable her to read common letters with her left eye and to see all larger objects with the right. Ware included that report also in the second and third edition of his treatise, which were published in 1804 and 1812, respectively, under the abridged title *Observations on the cataract and gutta serena*.

The subacute onset, the close temporal relationship of the two events, the intensity of the attacks (leading to almost complete paresis and complete visual loss within short time), the symptoms accompanying visual loss (dilated pupils rather than tabetic miosis, violent eye pain), the absence of signs of cerebral disease, and, finally, the partial recovery, all strongly argue against tabes (though other, rare complications of syphilis, a history of which was not mentioned by Ware, cannot be formally excluded) but strongly support a diagnosis of NMO in this patient.

Interestingly, the patient, a women of 45 years (the median age of onset of AQP4-IgG-positive NMO, which mainly affects women, is 40 years [1]), had been for many years *“subject to frequent returns of rheumatic affections in different parts of the body”*. Given that AQP4-IgG-positive NMO has been demonstrated to be frequently associated with connective tissue disorders such as systemic lupus erythematosus, Sjögren’s syndrome, or rheumatoid arthritis in a substantial number of cases [1, 59, 229], the latter fact does not argue against but rather supports a diagnosis of NMO. As a limitation, the exact nature of this patient’s rheumatic affliction remains elusive, since Ware does not provide us with more detail.

Ware, a pupil of Jonathan Wathen (1728–1808) and contemporary of Beer, is considered one of the founding fathers of modern ophthalmology in Great Britain [230–232]. He was admitted a Fellow of the Royal Society in 1802 – the first “bare oculist” in the history of the society. The *Dictionary of National Biography* underlines Ware’s contributions to establishing ophthalmology as a scientific subject in Britain: *“It is the peculiar merit of Wathen and of his pupil Ware that they elevated ophthalmic surgery from the degraded condition into which it had fallen. Originally a branch of general surgery, but always invaded by quacks, it fell into dishonest hands, from which the disinterested efforts of men like Ware first rescued it”* [230].

**Fig. 7:**
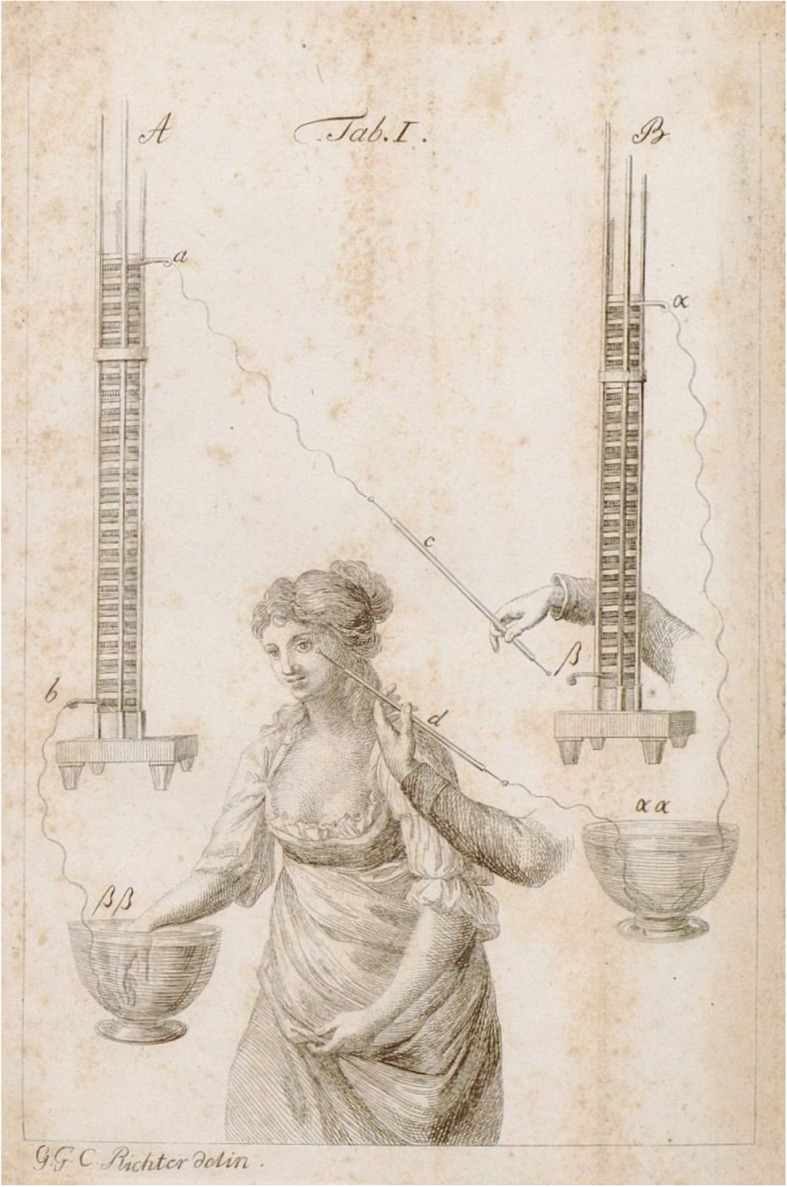
Galvanic treatment of an amaurotic women by use of two Volta columns, taken from Christian Heinrich Ernst Bischoff’s *Commentatio de usu Galvanismi in arte medica speciatim vero in morbis nervorum paralyticis* (1801)

However, electrotherapy – first applied by Christian Gottlieb Kratzenstein (1723–1795) and Johann Gottlob Krüger (1715–1759) in Germany in the 1740s and much promoted by John Wesley (1703–1791), the founder of Methodism, in Great Britain – which both Baillie and Ware commended to their patients, was itself prone to charlatanry. The period following Luigi Galvani’s (1737–1798) discovery in 1780 and the debate with Alessandro Volta (1745–1827), and especially the years around the turn of the century directly preceding the appearance of Ware’s report, had seen the publication of numerous books on the topic (e.g., Ritter 1800; Reinhold 1797/98; Grapengießer 1801, Augustin 1801, Struve 1802, Hellwag 1802, Sternberg 1803, Clarus 1802/3, Weber 1802, Eschke 1803, Reinhold 1803), and many of the therapeutic recommendations made at the time were insufficiently (criticised as *“grobempirisch”* as early as 1816 [233]) or not at all evidence-based. Figure 7 shows an apparatus used to treat amaurosis (taken from Christian Heinrich Ernst Bischoff’s [1781–1861] *Commentatio de usu galvanismi in arte medica speciatim vero in morbis nervorum paralyticis* in which he propagated the use of galvanism for treating neurological diseases, including paresis and amaurosis as well as rheumatic disorders [234]).

### Joseph Warner (1773): severe amaurosis and paraparesis

**Fig. 8:**
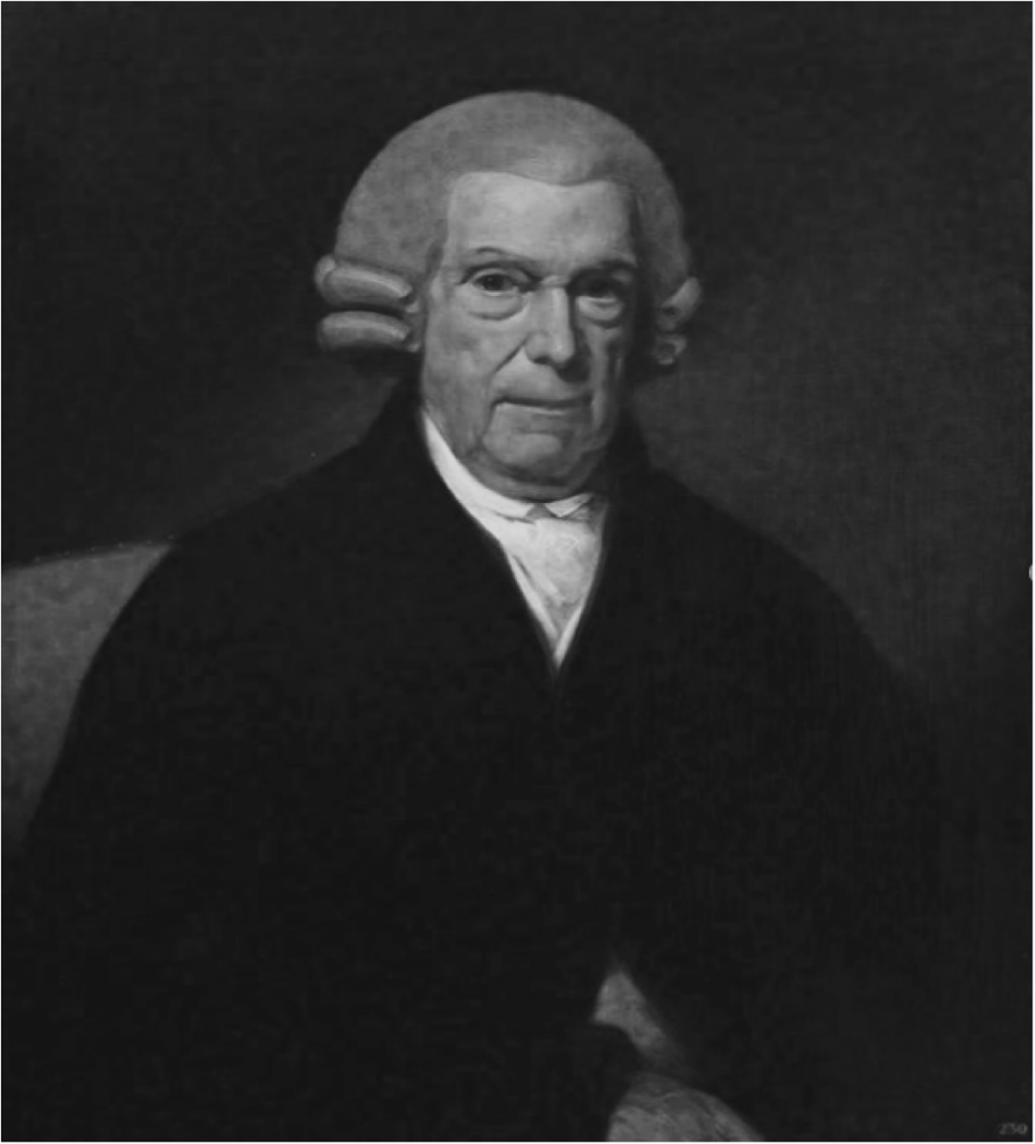
Joseph Warner (1717–1801)

In his *Description of the human eye, and its adjacent parts; together with their principal diseases, and the methods proposed for relieving them* (1773) [235], which Beer highly esteemed despite its brevity, and which Hirschberg considered – as he felt it was based on practical knowledge – the first useful ophthalmological textbook written in the English language, the Antigua-born Joseph Warner (1717–1801) (Fig. 8), then first physician to Guy’s Hospital (a position he held for 40 years) and Fellow of the Royal Society [7, 236], reported the following case of severe bilateral amaurosis with concurrent paraplegia but apparently no signs or symptoms of brain involvement:

*“In the instance of a young gentleman, whom I several times visited on a similar occasion, his defect of sight was accompanied by a palsy of the lower limbs (…) the fibres of the iris had no visible motion; the humours of the eyes preserved their natural transparency; his sight was not totally lost, but it was become so much impaired, as to be of little or no use to him; and it seemed to be affected in a degree pretty nearly equal to that of his legs and thighs.”*

This could well be the earliest description of a case of NMO known so far. The words “several times on a similar occasion” even suggest a relapsing course of disease. However, it is an obvious limitation that Warner specified neither whether the symptoms were of acute onset nor whether any other signs or symptoms of myelitis were present. Accordingly, considerable uncertainty regarding the exact diagnosis must remain.

Given its high prevalence, syphilis would be a potential differential diagnosis. It is therefore of particular interest that Warner, who related this case as an example for his more general observation of amaurosis not always (although mostly) being followed by mydriasis, gives us the following additional information: “*in [this patient’s] eyes the pupils were contracted to at least half their natural size*”. This could well be a very early description of syphilitic miosis, which would later be analysed in detail by Douglas Argyll Robertson (1837–1905), von Graefe’s famous Scotish pupil (cf. Argyll Robertson’s seminal 1869 articles on the topic [237, 238], in which, however, he acknowledges having learned about the phenomenon already at Robert Remak’s [1815–1865] clinic in Berlin and in which he also referrs to earlier descriptions by Romberg [to be found in vol. 1, dev. 2, of his *Lehrbuch der Nervenkrankheiten des Menschen*, first edn. 1840–1846 [239], English translation 1853 [240]], by Armand Trousseau [1801–1867] [see the revised chapter on locomotor ataxia included in the third volume of his *Clinique médicale de L’Hotel-Dieu dè Paris*, first edn. 1865 [241]; English translation 1867 [242]], and by Stellwag von Carion in his *Lehrbuch der Augenheilkunde* [first edition 1861/1862 [105]; English translation 1868]; see also [243, 244]).

On the other hand, disturbances in pupillary function, including miosis, have been repeatedly reported also in AQP4-IgG-positive NMO [245–249], and the periaqueductal area, believed by some authors to contain the mostly likely site of damage in patients with Argyll Robertson syndrome, is also a site of high AQP4 expression and, accordingly, not infrequently a site of inflammation in AQP4-IgG-positive NMO [9, 250]. Finally, brainstem involvement is also a common feature in MOG-IgG-positive NMO [10], and periaqueductal lesions have also been reported in this condition. The presence or absence of miosis thus might not be a sufficient criterion for distinguishing neurosyphilis and autoimmune NMO.

The relationship of neurosyphilis and NMO is complex. NMO was originally defined as a syndrome rather than a pathogenetically defined disease entity [5, 44]. In consequence, the presence of syphilis would per se not necessarily have precluded a diagnosis of NMO. As mentioned above, some of the patients included by Devic and Gault as cases of NMO in their disease-defining review [44] in fact had a history of syphilis. Whether this was a simple coincidence owing to the high prevalence of syphilis at that time (only a minority of patients affected by primary lues will later develop neurosyphilis) or whether NMO was caused by syphilitic lesions in these cases is unknown. Apart from tabetic degeneration of the optic nerves and posterior columns, neurosyphilis can cause spinal pachymeningitis, meningomyelitis, and spinal endarteritis, which have at least the potential of causing paraplegia (see also Erb’s 1902 London lecture *On spastic and syphilitic paralysis* [251]). Similarly, ON is counted among the possible (though very rare) complications of neurosyphilis. The incidence of neurosyphilis, one of the most common neurological diseases of the 18th and 19th century, has significantly declined since the introduction of penicillin as a treatment for lues. Neurosyphilis is now a very rare diagnosis. Accordingly, in recent decades only a few patients with NMO and syphilis have been reported in the Western medical literature, one of whom was tested for NMO-IgG but was negative for that marker [252, 253].

### Alexander (1827), Marcé (1845/1847) and Bradley (1818)

Mainly to avoid remonstration by the informed reader, who may be aware of these cases, but also for the sake of completeness, we would like to briefly mention three further, though more doubtful, reports of possible NMO that appeared in *The Lancet* in 1827; in a small local journal, the *Journal de la Section de médecine de la Société académique du département de la Loire-inférieure*, in 1847; and in a medical textbook entitled *On some varieties of spinal disease*, published in 1818.

The first report, published by John Alexander, a Manchester-based physician, describes the case of a 12-year-old girl [254]. While the title of the article (“On intermitting paraplegia, combined with amaurosis”) is highly suggestive of NMO, the case itself is very unusual in that the onsets of paraplegia and amaurosis were preceded or accompanied, respectively, by chorea-like disease, fits that “*assumed the appearance of epilepsy*” and “*occasional phrensy inducing her to run out of the house, and proceed until exhausted*”. Moreover, rubbing a little ointment in the neck, as a placebo, was followed by rapid recovery, rendering the diagnosis of a functional disorder at least conceivable. The term “spinal amaurosis” was not used. In the medical literature of the 19th century, “hysteric” amaurosis, paraplegia or tetraplegia is a recurrent topic. On the other hand, it should be noted that epileptic seizures as well as psychiatric symptoms have been reported both in AQP4-IgG-positive and in MOG-IgG-positive NMO, in particular in children [12–15].

The second report is part of a series of observations on *“myélites spontanées qui se sont sporadiquement manifestées à Nantes à dater des derniers mois de 1845”* by Germain-Auguste Marcé (1805–1859; see reference [255] for a biographical sketch) and describes the case of a male patient, an abbot, who, after exposure to cold and humidity, developed fever, spastic and painful sensorimotor paraparesis and, in addition, plegia of the left arm and right hand. The patient did not response to various treatment attempts, including bloodletting, vesicatories, strychnine and quinine. New sensory symptoms occurred about 2.5 months after onset, and at around 5 months after onset he experienced an acute attack which led to blindness in the left eye and complete tetraparesis, associated with slight dysarthria. The patient died a few days later. No autopsy was performed. During the entire duration of the disease there was no clinical evidence for supratentorial brain involvement, and *“les idées étaient parfaitement lucides”*. A diagnosis of inflammation of the spinal cord with concomitant visual loss was made. While the onset of both AQP4-IgG- and MOG-IgG-positive autoimmune NMO is not rarely preceded by acute infection, [1, 51] the fact that fever and sweating persisted for a considerable time throughout the course of disease is rather atypical and suggests a possible infectious aetiology in this case.

The third case is that of a 17-year-old girl who presented with visual deficiency, a mydriatic pupil with diminished light reaction, some weakness of the lower extremities, considerable pain when pressure was exerted on the third and fourth vertebrae, and vertigo [256]. However, the case is otherwise rather obscure and it is doubtful whether the patient had NMO. The author’s final recommendation, made as early as 1818, thus seems to be of more importance to us than the case itself: *“[it is necessary] both for the oculist and [the] general practicioner clearly to ascertain, in cases of dysopsia, whether the disease be not accompanied with constitutional symptoms dependent on some affection of the spine.”*

### Peltesohn (1886): early epidemiological data on the coincidence of myelitis and optic neuritis

We would like to draw the reader’s attention to a work which seems particularly noteworthy by virtue of being the first ‘epidemiological’ work on NMO. In an article series – an excerpt from his dissertation – on the causes and course of optic nerve atrophy, published in three parts in *Zentralblatt für praktische Augenheilkunde* in 1886 [126–128], the German ophthalmologist Nathan Peltesohn (1862–1942) related the case of a 36-year-old merchant with cervical myelitis, resulting in spastic tetraparesis, hyperreflexia, paraesthesia, girdle band sensation, and urinary and erectile dysfunction. The patient developed complete bilateral amaurosis; ophthalmoscopy revealed optic nerve atrophy. However, similar to some patients reported by Devic and Gault [44], the anamnestic information suggested a history of lues. An infectious aetiology can thus not be excluded. Interestingly, Peltesohn’s patient was clinically examined by no lesser figures than Julius Hirschberg, still known to many as the author of the monumental *Geschichte der Augenheilkunde*, published in nine volumes between 1899 and 1918 (English edition 1982–1994), and the psychiatrist and neurologist Emanuel Mendel (1839–1907), teacher to Max Bielschowsky (1869–1940) and Sigmund Freud (1856–1939). In this work, Peltesohn included for the first time statistical data (from Hirschberg’s department) on the prevalence of myelitis in patients with optic nerve atrophy. Among 248 patients with “neuritic atrophy” of the optic nerve(s), i.e. atrophy following ON, only two also (0.8%) had myelitis; and among 248 patients with non-neuritic, “simple atrophy” of the optic nerve, two (0.8%) had “chronic myelitis” (all patients were seen between 1880 and 1885). Dr. Peltesohn, who continued to practice privately in Hamburg after withdrawal of the accreditation of Jewish doctors with the statutory health insurance companies in 1933, was murdered by the Nazis, together with his two sisters, in 1942, aged 78.

### François Boissier de Sauvages de Lacroix: ‘Amaurose rachialgique’ (1763)

In the final two sections we return briefly to nomenclature (an important topic, since it helps to structure medical knowledge: ‘[w]hat is a disease before it gets a name?’ [T. Jock Murray, Multiple Sclerosis: The History of a Disease, New York, 2005]) by referring to a curious contribution by François Boissier de Sauvages de Lacroix’s (1706–1776) and, finally, by discussing the reasons for the change in terminology that was to occur at the turn to the twentieth century.

In 1763, de Sauvage (botanical author citation: Sauv), inspired by Linné’s ground-breaking works – *Systema Naturae* (which, despite all the known limitations resulting from its artificial nature, marks the beginning of modern zoological nomenclature) had just appeared in 1758 – and Sydenham’s attempts to use a ‘binary’ or ‘binomial’ system also for the classification of diseases, published his famous and monumental *Nosologia methodica* in ten volumes. Further nosological compendia were published by William Cullen (1710–1790), Whytt’s predecessor in Edinburgh, between 1769 and 1785, and by Jean Baptiste Théodore Baumès (1756–1828) in 1806.

Interested in whether any of these authors was aware of amaurosis spinalis (a binomial term in itself, consisting of species and epithet), we came indeed across a very similar term in de Sauvage’s nosology, namely that of *‘amaurose rachialgique’*. Considering that Hippocrates had referred to myelitis as ‘lumbago’ and Brera as ‘rachialigitis lumbalis’, ‘amaurose rachialgique’ clearly evokes the concept of ‘spinal amaurosis’.

The term would later be adopted by Jean Baptiste Théodore Baumès (1756–1828), who is now mainly remembered for his iatro-chemical ideas, *i.e.* for pointing to the connection between medicine and chemistry (*Essai d’un système chimique de la science de l’homme* 1798). In both his *Fondemens de la science méthodique des maladies* (1801–1802) [257] and his *Traité élémentaire de nosologie* (1806) [258] we find the following entry: *“Amaurose rachialgique; Sauv., cl. VI, ord. I, gen. IV* (should be gen. V according to our edition of de Sauvages),* esp. 14”.* However, when looking up the lemma ‘rachialgia’, there is no explicit reference to spinal inflammation, but exclusively to pain related to the spine: *“Syn. Rachialgie (de rachis, épine du dos; et algeia, douleur); Sauvages, cl. VII, doleurs ; ord.. V, externes et des membres ; gen. XXIX.– Sagar, cl. IV, doleurs; ord. V, locales des parties externes ; gen. XXVII”.* Although myelitis can certainly be associated with local spinal pain, it remains elusive what exact conditions de Sauvages had in mind when creating that expression. We could not find any other instance for the use of that peculiar expression in the medical literature, rendering it almost a *hapax legomenon*.

The most famous British nosological work of the 18th century is certainly Cullen’s *Synopsis Nosologiae Methodicae* (1769–1785), an English translation of which appeared in 1800 under the title *Nosology; or, a systematic arrangement of diseases, by classes, orders, genera, and species* (1800) [259]. Interestingly, Cullen knows a category [“genus”] termed *amaurosis spasmodica*, which he classifies under class IV [*locales*], order I [*dysæsthesiæ*]. *Spasmi* in turn he lists in class II [*neuroses*] as order III, in which he defines a subcategory termed *tetanus* [“spasmodic rigidity of several muscles”] (not to be confused with the modern usage of this term). However, Cullen refers only to hemiplegia [*tetanus hemiplegicus*] here, not to paraplegia or to spinal cord damage. Although he explicitly made use of the work of his predecessor, Cullen did not adopt de Sauvage’s term *‘amaurose rachialgique’.*

## From ‘spinal amaurosis’ to ‘neuromyelitis optica’

Given the clear and distinct character of the term ‘spinal amaurosis’, which follows an Aristotelian rule of definition, which requires that *„definitio fiat per genus proximum et differentiam specificam*”, it is – at least at first glance – not easy to understand why it would be replaced by ‘neuromyelitis optica’, a highly artificial term that purists might regard as linguistic barbarism [260], later on. All the more so because the term ‘neuromyelitis’ – though this may not have been known to Devic and Gault – had been in use before, with different meanings. In total, we came across at least six different usages of the term, listed here in chronological order:

i. ‘Nevromyelitis’ denoting inflammation of the *pulpa nervorum* as opposed to inflammation of the *vagina nervorum* (‘nevrilemmatitis’); in this sense the term was probably first used by Johann Valentin Hildenbrand (1763–1818) in his famous *Institutiones practico-medicae* (published posthumously by his son in 1822) and later adopted [261–263] but also criticised [264–270];
ii. ‘neuromyelitis’ as a synonym of ‘inflammation of the spinal cord’, as defined in the 1836 edition of the prestigious *Dictionnaire de l’Académie Française* and later adopted by others (see, for example, reference [271]: *“Ha recibido los nombres de espinitis, myelitis, neuromyelitis, notomyelitis”* [1843]);
iii. ‘neuromyelitis’ as a synonym of ‘inflammation of the vertebra’ (1875) [272];
iv. ‘neuromyelitis’ as a synonym of ‘spinal nerve disease’, as used by Allbutt in an address entitled *On the Surgical Aids to Medicine* delivered at the inaugural meeting of the Midland Medical Society on 19th October 1881 [273];
v. ‘neuromyelitis’ (or ‘ascending neuromyelitis’ or ‘neuromyelitis hyperalbumenotica’ [274]) as a synonym of ‘Landry’s syndrome’ or ‘Guillain-Barre syndrome’, as still listed in the World Health Organization’s International Classification of Diseases (see ICD 61.0);
vi. ‘neuromyelitis’ as a component and as an abbreviation of ‘neuromyelitis optica’ (1894) [44] or, most recently, ‘neuromyelitis optica spectrum disorders’ [250].

We believe that several factors determined the rapid change in nomenclature that took part at the turn of the century:

1. As mentioned above, a shift in use of the term ‘spinal amaurosis’ towards being employed as a synonym of ‘tabes dorsalis with amaurosis’ took place in the second half of the 19th century. This parallels the growing interest in tabes following accumulating evidence for its being caused by syphilis, especially after the appearance of Fournier’s *De l’ataxie locomotrice d’origine syphilitique* in 1875/1876 [275, 276] and, a fortiori, after support for Fournier’s hypothesis from Erb [277] (in disagreement with Leyden), Vulpian (dissenting in that regard with his former co-author on tabes, Charcot) [278] and Gowers [279]. This created a need for a new term that would better distinguish the two conditions.
2. This shift may be related to the rise in prevalence of neurosyphilis over the course of the 19th century suspected by some authors [280] or, at least, be a result of the much higher prevalence of tabetic amaurosis than of NMO at that time. While NMO remains a rare disease to the present day, syphilis was highly prevalent far into the 20th century. The Royal Commission on Venereal Diseases in 1916 concluded that the disease’s prevalence *“cannot [be] below 10 per cent of the whole population in the large cities”* [281]. The high number of tabetic cases may thus have simply overshadowed or eclipsed the previous knowledge about rare cases of non-tabetic spinal cord and optic nerve damage reported throughout the 19th century. This may be true especially for neurology, a clinical discipline that was just emerging (Charcot’s chair at the Salpêtrière, the first one worldwide dedicated specifically to diseases of the nervous system, was established only in 1882) and in the process of setting up its own discrete nomenclature. Many physicians dedicating themselves to diseases of the nervous system at the end of the nineteenth century may simply not have been aware of the previous ophthalmological literature on spinal amaurosis, especially that from the first half of the century, as is evident, for example, from the above-cited statements by Erb, Allbutt and Gowers.
3. The fact that the term ‘spinal amaurosis’ places the emphasis on amaurosis, which may not appropriately reflect both the great disease burden inflicted by spinal disease as well as the greater interest neurologists may attach to myelitis than to amaurosis in general and in particular may have attached to that syndrome at a time when great progress was being made in elucidating the physiology and pathology of the spinal cord.
4. Finally, the wording ‘spinal amaurosis’ can be understood as implying a causal relationship. However, the notion of spinal cord disease causing amaurosis (*e.g.* by procuring damage to the spinal sympathetic nerves) in patients with ‘spinal amaurosis’ was not proven and, accordingly, not accepted by all, including such influential figures as Erb, Allbutt and Gowers.

## Discussion

Until very recently, most review articles and book chapters on NMO let the history of that syndrome begin with Devic and Gault’s – indeed seminal – review *De la neuromyélite optique aiguë* [44], which appeared in 1894, and a brief reference to patients with acute myelitis and optic nerve damage by Thomas Clifford Allbutt in his 1870 article on the ophthalmoscopic signs of spinal cord disease was considered by many the first mention of a patient with possible NMO in the medical literature [94].

However, as we were able to demonstrate in a recent series of articles, Devic and Gault as well as Allbutt overlooked numerous earlier case of NMO, probably owing to the limited bibliographic means of the time. These reports – one published in *The Lancet* in 1865 [93] by the famous British neuroanatomist and neurophysiologist Jacob August Lockhart Clarke, eponym for the posterior thoracic nucleus or Clarke’s column, one by the British physician Christopher Mercer Durrant in 1850 in the *Provincial Medical and Surgical Journal* [92], the precursor of the *BMJ*, one by the Genoese physician Giovanni Battista Pescetto in a local Italian journal in 1844 [31], and one by Edward Hocken, a young but already eminent British ophthalmologist, in *The Lancet* in 1841 – all clearly precede Allbutt’s article, demonstrating that the concurrence of amaurosis and spinal cord damage so characteristic for NMO had caught the attention of physicians much earlier than previously thought.

The cases re-presented here, which date back to the late 18th and early 19th century – and thus mostly antedate even the cases described by Clarke, Durrant, Pescetto and Hocken – provide additional evidence for the latter notion. In addition, we re-present the concept of spinal amaurosis, which, although the immediate precursor of NMO, was virtually forgotten. Of note, this concept was introduced not by neurologists but by ophthalmologists, who were, as shown here, the first to take note of the syndrome. The term ‘spinal amaurosis’ (and its variants) can be traced back, as demonstrated here, to the first half of the 19th century and remained present in the medical literature until the end of that century, from which time – in parallel with the establishment of neurology as a new discipline with its own, distinct nomenclature – it was gradually superseded by ‘NMO’ and its variants (‘neuro-myélite optique aiguë’, first proposed by Devic and Gault 1894 [44, 133, 137]; ‘acute optic neuromyelitis’, anonymous 1903 [282, 283]; ‘neuromyelitis optica’, Stransky 1904 [284]) and by corresponding eponymous designations (‘maladie de Devic’, first proposed by Peppo Acchiote 1907 [285–287]; ‘Devic[‘s] disease’; ‘Devic[‘s] syndrome’; ‘Devic-Erkrankung’; ‘Morbus Devic’).

While the simultaneous occurrence of optic nerve and spinal cord damage was, as shown here, already noted in the late 18th and 19th century, the exact nature of the pathogenic relationship between the two affections remained elusive. While some authors believed that damage to the spinal sympathetic nerve might impair the blood supply of the optic nerve, others believed that amaurosis resulted from ascending meningitis, originating in the spinal cord and propagated to the optic nerves *per continuitatem*, or wondered about the possible existence of an unrecognized anatomical connection between the spinal cord and optic nerves. Only a few authors, among them Gowers and Erb, but also Gault and Devic, would (explicitly or implicitly) dismiss the idea of a direct link and maintain the view that the two sites were independently affected by a common causal factor. It would take more than two centuries for more light to be shed on the pathogenesis of NMO, when a role for antibodies was suggested based on histopathological findings by Lucchinetti and colleagues in 1999 [57] and when finally aquaporin-4, the most abundant water channel in the CNS, was revealed as target antigen in the majority of patients with NMO by Lennon and colleagues in 2004/2005 [2, 47]. Recent studies suggesting differences in AQP4 expression levels and isoform composition between CNS regions [288] as well as the hypothesis of a higher permeability or vulnerability of the blood–brain barrier in some areas of the optic nerve and spinal cord [289–293] provide a potential rationale for the predominant involvement of the optic nerves and spinal cord in this disease, which had been a riddle for more than 200 years. Moreover, pathogenic autoantibodies to MOG have been identified in a subset of patients with AQP4-IgG-negative NMO [10, 50–53]. In some AQP4-IgG- and MOG-IgG-negative NMO patients a beneficial effect of plasma exchange has been reported, suggesting a humoral pathogenesis and the presence of (so-far unknown) autoantibodies. Finally, rare cases caused by paraneoplastic autoimmunity (e.g. to CV2/CRMP5 [294]) or related to neurosarcoidosis have been identified. The pathogenetic heterogeneity in NMO suggested by these findings made changes in nomenclature and nosology inevitable: NMO is no longer considered a single disease but – well in line with Devic and Gault's original definition – rather viewed as a clinical or clinicoradiological syndrome or phenotype shared by two or more pathogenetically distinct diseases. AQP4-IgG-positive NMO and MOG-IgG-positive NMO are now recognised by most authors as entities in their own right distinct from classic MS. Accordingly, diagnostic criteria different from those used to diagnose MS [295] have been proposed [54, 250].

The origins of the concept of spinal amaurosis fall into the ‘ophthalmological renaissance’ in early 19th century France that followed the temporary yet substantial decline of ophthalmology in the aftermath of the French revolution and which was driven mainly by the immigration of ophthalmologists from abroad, such as Sichel, and the recognition of the important progress ophthalmology had meanwhile made outside France [295]. However, as shown here, very early cases of possible NMO can also be found in the works of physicians based in other European countries, including the United Kingdom, and the term ‘spinal amaurosis’ was soon adopted throughout the continent.

Intriguingly, spinal amaurosis or NMO, remained a topic of interest at some centres from the 19th century, with some interruptions, right up to the present time: Pétrequin, born in Villeurbanne, for many years held the prestigious position of *“chirurgien-major”* at the Hôtel-Dieu de Lyon, the very city that later became linked forever with NMO by the seminal works of Devic and Gault [44, 133, 137], which would appear 18 years after Pétrequin’s death. Since then, the Lyon school of neurology has maintained a strong interest in NMO (e.g., [296–301]), partly driven by Christian Confavreux (1949–2013), a world-renowned specialist in MS, who married into the Devic family [302]. Chelius, on the other hand, who contributed greatly to popularizing the new concept of ‘spinal amaurosis’, was a graduate of the University of Heidelberg, where he was appointed professor of general surgery and ophthalmology in 1817. Chelius, rated by August Hirsch in his *Biographisches Lexikon der hervorragenden Aerzte aller Zeiten und Völker* as one of the most renowned and esteemed physicians and surgeons of his time in Europe [303], is not only considered the founding father of the Heidelberg school of ophthalmology but was instrumental in laying the foundations for the later high reputation of the Heidelberg medical school [304]. Around 25 years after Chelius’ book appeared in print, Wilhelm Erb became a professor at Heidelberg, where he later – after an intermezzo in Leipzig – would succeed Nikolaus Friedreich (1825–1882), eponym of Friedreich’s ataxia, as head of department. It was in 1879 that Erb published the first German report on a patient with NMO, which is still considered the most detailed clinical case study of its time [97]. A short time later, a case of bilateral optic neuritis and longitudinally extensive transverse myelitis (accompanied by involvement of the medulla oblongata) was reported by Herman Jakob Knapp (1832–1911) [305]. Knapp had been encouraged by Robert Bunsen (1811–1899), the father of spectral analysis, to study medicine under Hermann Ludwig Ferdinand von Helmholtz (1821–1894) in Heidelberg and had been made professor of ophthalmology in 1860. Later, in 1896, Theodor von Leber’s doctoral student Karl Katz published his dissertation on NMO in Heidelberg [136]. Leber, eponym of Leber’s hereditary optic neuropathy, a former pupil of Helmholtz and assistant of Knapp, had been made head of the Heidelberg department of ophthalmology in 1890. Nowadays, the Neuroimmunology Group at the University Hospital Heidelberg attempts to honour that heritage by dedicating much of its work to improving the diagnosis and treatment of NMO as well as deepening our understanding of the pathogenesis of this rare syndrome (e.g., [1, 3, 4, 10, 49, 51–53, 306–309]).

## Conclusion

The recent discovery of AQP4-IgG in patients with NMO has revived interest in the co-occurrence of eye disorders and myelitis. We believe the time has come to do justice to those who were the first to report on this rare yet intriguing syndrome by acknowledging their achievements and underlining their position in the history of medicine. The reports on amaurosis in patients with spinal cord inflammation by Ware, Baillie, Sichel, Carron du Villard and Pétrequin, re-presented here, are the earliest descriptions of possible NMO in the Western medical literature and thus deserve to be remembered. In addition, we point to the concept of ‘spinal amaurosis’, a long-forgotten precursor of the concept of NMO, and emphasise the important role played by ophthalmologists in first describing the syndrome. We hope that our findings may kindle further research into the early history of NMO.

## Data Availability

The datasets generated and/or analysed during the current study are not publicly available but are available from the corresponding author upon reasonable request.

## Abbreviations

AQP4: Aquaporin-4
CNS: Central nervous system
IgG: Immunoglobulin G
MOG: Myelin oligodendrocyte glycoprotein
MS: Multiple sclerosis
NMO: Neuromyelitis optica
NMOSD: NMO spectrum disorders

## References

[1] Jarius S, Ruprecht K, Wildemann B, Kuempfel T, Ringelstein M, Geis C, Kleiter I, Kleinschnitz C, Berthele A, Brettschneider J (2012). Contrasting disease patterns in seropositive and seronegative neuromyelitis optica: A multicentre study of 175 patients. J Neuroinflammation.

[2] Lennon VA, Wingerchuk DM, Kryzer TJ, Pittock SJ, Lucchinetti CF, Fujihara K, Nakashima I, Weinshenker BG (2004). A serum autoantibody marker of neuromyelitis optica: distinction from multiple sclerosis. Lancet.

[3] Jarius S, Paul F, Franciotta D, Waters P, Zipp F, Hohlfeld R, Vincent A, Wildemann B (2008). Mechanisms of disease: aquaporin-4 antibodies in neuromyelitis optica. Nat Clin Pract Neurol.

[4] Jarius S, Wildemann B (2010). AQP4 antibodies in neuromyelitis optica: diagnostic and pathogenetic relevance. Nat Rev Neurol.

[5] Jarius S, Wildemann B (2013). The history of neuromyelitis optica. J Neuroinflammation.

[6] von Walther PF (1846). Kataraktologie. J der Chirurgie und Augenheilkunde.

[7] Hirschberg J (1899). Geschichte der Augenheilkunde, 2nd edition.

[8] Sichel J (1837). Traité de l'ophthalmie, la cataracte et l'amaurose.

[9] Pittock SJ, Weinshenker BG, Lucchinetti CF, Wingerchuk DM, Corboy JR, Lennon VA (2006). Neuromyelitis optica brain lesions localized at sites of high aquaporin 4 expression. Arch Neurol.

[10] Jarius S, Kleiter I, Ruprecht K, Asgari N, Pitarokoili K, Borisow N, Hummert MW, Trebst C, Pache F, Winkelmann A (2016). MOG-IgG in NMO and related disorders: a multicenter study of 50 patients. Part 3: Brainstem involvement - frequency, presentation and outcome. J Neuroinflammation.

[11] Wingerchuk DM, Hogancamp WF, O'Brien PC, Weinshenker BG (1999). The clinical course of neuromyelitis optica (Devic's syndrome). Neurology.

[12] Hamid SHM, Whittam D, Saviour M, Alorainy A, Mutch K, Linaker S, Solomon T, Bhojak M, Woodhall M, Waters P (2018). Seizures and Encephalitis in Myelin Oligodendrocyte Glycoprotein IgG Disease vs Aquaporin 4 IgG Disease. JAMA Neurol.

[13] Lotze TE, Northrop JL, Hutton GJ, Ross B, Schiffman JS, Hunter JV (2008). Spectrum of Pediatric Neuromyelitis Optica. Pediatrics.

[14] McKeon A, Lennon VA, Lotze T, Tenenbaum S, Ness JM, Rensel M, Kuntz NL, Fryer JP, Homburger H, Hunter J (2008). CNS aquaporin-4 autoimmunity in children. Neurology.

[15] Ogawa R, Nakashima I, Takahashi T, Kaneko K, Akaishi T, Takai Y, Sato DK, Nishiyama S, Misu T, Kuroda H (2017). MOG antibody-positive, benign, unilateral, cerebral cortical encephalitis with epilepsy. Neurol Neuroimmunol Neuroinflamm.

[16] Carnero Contentti E., Leguizamón F., Hryb J.P., Celso J., Di Pace J.L., Ferrari J., Knorre E., Perassolo M.B. (2016). Neuromyelitis optica: Association with paroxysmal painful tonic spasms. Neurología (English Edition).

[17] Hamid S, Elsone L, Mutch K, Hunt DP, Murray K, Reid JM, Jacob A (2015). Tonic spasms and short myelitis in an elderly woman-unique onset of neuromyelitis optica. Pract Neurol.

[18] Kim SM, Go MJ, Sung JJ, Park KS, Lee KW (2012). Painful tonic spasm in neuromyelitis optica: incidence, diagnostic utility, and clinical characteristics. Arch Neurol.

[19] Usmani N, Bedi G, Lam BL, Sheremata WA (2012). Association between paroxysmal tonic spasms and neuromyelitis optica. Arch Neurol.

[20] Kim W, Kim SH, Nakashima I, Takai Y, Fujihara K, Leite MI, Kitley J, Palace J, Santos E, Coutinho E (2012). Influence of pregnancy on neuromyelitis optica spectrum disorder. Neurology.

[21] Bourre B, Marignier R, Zephir H, Papeix C, Brassat D, Castelnovo G, Collongues N, Vukusic S, Labauge P, Outteryck O (2012). Neuromyelitis optica and pregnancy. Neurology.

[22] Deval C: Traité de l'amaurose ou de la goutte-sereine. Ouvrage contenant des faits nombreux de guérison de cette maladie, dans des cas de cécité complète. Paris Victor Masson; 1851.

[23] Scarpa A (1801). Saggio d’osservazioni e d’esperience sulle principali malattie degli occhi.

[24] Keeler R, Singh AD, Dua HS (2013). Of Fathers and Sons: Antonio Scarpa (1752–1832). Brit J Ophthalmol.

[25] Carron du Villards CJF: Lettre de M. Carron du Villards à M. Pétrequin Annales d'Oculistique et Gynécologie 1838, 1:87–90.

[26] Carron du Villards CJF (1838). Guide pratique pour l'etude et le traitement des maladies des yeux.

[27] Fernandez JS: Notice sur Carron du Villards. Annales d’Oculistique 1889, 101 (14^e^ Série, T. 1, 1^e^ et 2^e^ Iivraisons):12–28.

[28] Santos Fernandez J: Notice sur Carron du Villards. Annales d'Oculistique 1889, 14° Série, T. 1, 1e et 2e livraisons:11–28.

[29] Hirsch A, Wernich A (1884). Biographisches Lexikon der hervorragenden Aerzte aller Zeiten und Völker. Band 1.

[30] Otterburg SJ (1841). Ein Beitrag zur Geschichte der Medizin und ein Wegweiser für deutsche Ärzte.

[31] Jarius S, Wildemann B (2012). 'Noteomielite’ accompanied by acute amaurosis (1844). An early case of neuromyelitis optica. J Neurol Sci.

[32] Bonnan M, Valentino R, Olindo S, Mehdaoui H, Smadja D, Cabre P (2009). Plasma exchange in severe spinal attacks associated with neuromyelitis optica spectrum disorder. Mult Scler.

[33] Magana SM, Keegan BM, Weinshenker BG, Erickson BJ, Pittock SJ, Lennon VA, Rodriguez M, Thomsen K, Weigand S, Mandrekar J, et al. Beneficial Plasma Exchange Response in Central Nervous System Inflammatory Demyelination. Arch Neurol. 2011. 10.1001/archneurol.2011.34 PMC3134547 21403003

[34] Kleiter Ingo, Gahlen Anna, Borisow Nadja, Fischer Katrin, Wernecke Klaus-Dieter, Wegner Brigitte, Hellwig Kerstin, Pache Florence, Ruprecht Klemens, Havla Joachim, Krumbholz Markus, Kümpfel Tania, Aktas Orhan, Hartung Hans-Peter, Ringelstein Marius, Geis Christian, Kleinschnitz Christoph, Berthele Achim, Hemmer Bernhard, Angstwurm Klemens, Stellmann Jan-Patrick, Schuster Simon, Stangel Martin, Lauda Florian, Tumani Hayrettin, Mayer Christoph, Zeltner Lena, Ziemann Ulf, Linker Ralf, Schwab Matthias, Marziniak Martin, Then Bergh Florian, Hofstadt-van Oy Ulrich, Neuhaus Oliver, Winkelmann Alexander, Marouf Wael, Faiss Jürgen, Wildemann Brigitte, Paul Friedemann, Jarius Sven, Trebst Corinna (2015). Neuromyelitis optica: Evaluation of 871 attacks and 1,153 treatment courses. Annals of Neurology.

[35] Kleiter I, Gahlen A, Borisow N, Fischer K, Wernecke KD, Hellwig K, Pache F, Ruprecht K, Havla J, Kümpfel T (2018). Apheresis therapies for NMOSD attacks. A retrospective study of 207 therapeutic interventions. Neurol Neuroimmunol Neuroinflamm.

[36] Risse GB (1979). The renaissance of bloodletting: a chapter in modern therapeutics. J Hist Med Allied Sci.

[37] Guiart J (1937). Joseph-Pierre-Éléonord Pétrequin.

[38] Pétrequin JE (1838). Revue ophthalmologique de l’Hotel-Dieu de Lyon. Gazette medicale de Paris.

[39] Pétrequin JE (1838). Nouvelles remarques sur l’opération de cataracte par abaissement.

[40] Pétrequin JE (1838). Nouvelles remarques sur l’operation de la Cataracte par abaissment. Annales d’Oculistique et de Gynécologie.

[41] Pétrequin JE (1845). Mélanges de chirurgie, ou Histoire médico-chirurgicale de l'Hôtel-Dieu de Lyon, depuis sa fondation jusqu'à nos jours, avec l'Histoire spéciale de la syphilis dans cet hospice.

[42] Pétrequin JE (1877). Chirurgie d'Hippocrate.

[43] Pétrequin JE (1841). Noveau traité de l'amaurose ou goutte-sereine.

[44] Gault F (1894). De la neuromyélite optique aiguë. Thèse. Faculté de Médecine et de Pharmacie.

[45] Pétrequin JE (1840). Nouveau traité de l'amaurose ou goutte-sereine, considérée au point de vue clinique, avec des recherches nouvelles sur le méthodes spéciales de traitement qui conviennent a ses différentes espèces. Annales de la Société de Sciences Naturelles, de Bruges, année 1840–1841.

[46] Pétrequin JE (1838). Nouvelles recherches sur l'action thérapeutique de la noix vomique et de ses préparations dans les affections paralytiques. Gazette Medicale de Paris.

[47] Lennon VA, Kryzer TJ, Pittock SJ, Verkman AS, Hinson SR (2005). IgG marker of optic-spinal multiple sclerosis binds to the aquaporin-4 water channel. J Exp Med.

[48] Jarius S, Franciotta D, Bergamaschi R, Wright H, Littleton E, Palace J, Hohlfeld R, Vincent A (2007). NMO-IgG in the diagnosis of neuromyelitis optica. Neurology.

[49] Paul F, Jarius S, Aktas O, Bluthner M, Bauer O, Appelhans H, Franciotta D, Bergamaschi R, Littleton E, Palace J (2007). Antibody to aquaporin 4 in the diagnosis of neuromyelitis optica. PLoS Med.

[50] Mader S, Gredler V, Schanda K, Rostasy K, Dujmovic I, Pfaller K, Lutterotti A, Jarius S, Di Pauli F, Kuenz B (2011). Complement activating antibodies to myelin oligodendrocyte glycoprotein in neuromyelitis optica and related disorders. J Neuroinflammation.

[51] Jarius S, Ruprecht K, Kleiter I, Borisow N, Asgari N, Pitarokoili K, Pache F, Stich O, Beume LA, Hummert MW (2016). MOG-IgG in NMO and related disorders: a multicenter study of 50 patients. Part 2: Epidemiology, clinical presentation, radiological and laboratory features, treatment responses, and long-term outcome. J Neuroinflammation.

[52] Jarius S, Ruprecht K, Kleiter I, Borisow N, Asgari N, Pitarokoili K, Pache F, Stich O, Beume LA, Hummert MW (2016). MOG-IgG in NMO and related disorders: a multicenter study of 50 patients. Part 1: Frequency, syndrome specificity, influence of disease activity, long-term course, association with AQP4-IgG, and origin. J Neuroinflammation.

[53] Pache F, Zimmermann H, Mikolajczak J, Schumacher S, Lacheta A, Oertel FC, Bellmann-Strobl J, Jarius S, Wildemann B, Reindl M (2016). MOG-IgG in NMO and related disorders: a multicenter study of 50 patients. Part 4: Afferent visual system damage after optic neuritis in MOG-IgG-seropositive versus AQP4-IgG-seropositive patients. J Neuroinflammation.

[54] Jarius S, Paul F, Aktas O, Asgari N, Dale RC, de Seze J, Franciotta D, Fujihara K, Jacob A, Kim HJ (2018). MOG encephalomyelitis: international recommendations on diagnosis and antibody testing. J Neuroinflammation.

[55] Reindl Markus, Jarius Sven, Rostasy Kevin, Berger Thomas (2017). Myelin oligodendrocyte glycoprotein antibodies. Current Opinion in Neurology.

[56] Misu T, Fujihara K, Kakita A, Konno H, Nakamura M, Watanabe S, Takahashi T, Nakashima I, Takahashi H, Itoyama Y (2007). Loss of aquaporin 4 in lesions of neuromyelitis optica: distinction from multiple sclerosis. Brain.

[57] Lucchinetti CF, Mandler RN, McGavern D, Bruck W, Gleich G, Ransohoff RM, Trebst C, Weinshenker B, Wingerchuk D, Parisi JE, Lassmann H (2002). A role for humoral mechanisms in the pathogenesis of Devic's neuromyelitis optica. Brain.

[58] Levy M, Wildemann B, Jarius S, Orellano B, Sasidharan S, Weber MS, Stuve O (2014). Immunopathogenesis of neuromyelitis optica. Adv Immunol.

[59] Jarius S, Wildemann B, Paul F (2014). Neuromyelitis optica: clinical features, immunopathogenesis and treatment. Clin Exp Immunol.

[60] Lucchinetti CF, Bruck W, Lassmann H (2004). Evidence for pathogenic heterogeneity in multiple sclerosis. Ann Neurol.

[61] Jarius S, Konig FB, Metz I, Ruprecht K, Paul F, Bruck W, Wildemann B (2017). Pattern II and pattern III MS are entities distinct from pattern I MS: evidence from cerebrospinal fluid analysis. J Neuroinflammation.

[62] Sichel J (1840). Über die Augenentzündungen, den grauen und schwarzen Staar, deutsch bearbeitet von Theodor Gross.

[63] Sichel J (1839). Tratado de la oftalmia, catarata y amaurose; traducido del francés por José Zurita y José Bartorelo.

[64] Breßler (Bressler) H (1840). Die Krankheiten des Kopfes und der Sinnesorgane. Verlag der Voss'schen Buchhandlung.

[65] Chelius MJ (1843). Handbuch der Augenheilkunde.

[66] Himly K (1843). Die Krankheiten und Missbildungen des menschlichen Auges und deren Heilung.

[67] Vidal AT (1840). Traité de pathologie externe et de médecine opératoire.

[68] Guérineau J-D: Du diagnostic des maladies des yeux à l'aide de l'ophthalmoscope et de leur traitement. Par. Paris: P. Asselin , Gendre Et Successeur De Labé; 1860.

[69] Jarius S, Wildemann B (2014). 'Spinal amaurosis' (1841). On the early contribution of Edward Hocken to the concept of neuromyelitis optica. J Neurol.

[70] Hocken EO (1841). Illustrations on the pathology and treatment of amaurosis. Lancet.

[71] Hocken EO (1841). On the diagnosis, pathology, and treatment of amaurosis. Lancet.

[72] Hocken Edward (1841). ILLUSTRATIONS OF THE PATHOLOGY, DIAGNOSIS, AND TREATMENT OF OPHTHALMIC AFFECTIONS. The Lancet.

[73] Hocken EO (1842). Illustrations of the pathology, diagnosis, and treatment of ophthalmic affections. Lancet.

[74] Hocken E: Illustrations of the Pathology and Treatment of the Amauroses. Amaurosis from Affections of the Spinal Cord or its Membranes. London Medical Gazette 1841, 707. - XXVIII. New Series. Vol. II. For the session 1840–41.:499–504.

[75] von Walther FP (1841). Die Lehre vom schwarzen Staar und seine Heilart. Pathologie und Therapie der Amaurose. J der Chirurgie und Augenheilkunde.

[76] Ritter von Arlt CF: Die Krankheiten des Auges. Prag: Credner; 1856.

[77] von Graefe: Vorträge aus dessen Klinik über Amblyopie und Amaurose, mitgetheilt von Dr. Engelhardt. Klinische Monatsblätter für Augenheilkunde 1865, 3:131ff.

[78] von Graefe A: Clinical lectures on amblyopia and amaurosis. Boston; 1866.

[79] Liebreich R (1863). Atlas d'ophthalmoscopie; l'etat normal et les modifications pathologiques.

[80] Anonymous (1867). Reports of medical and surgical practice in the hospitals of Great Britain. National hospital for the epileptic and paralysed. Notes on cases of diseases of the nervous system (Under the care of Dr. Hughlings Jackson). Brit Med J.

[81] Weiß (Weiss) LS (1837). Die Augenheilkunde und die Lehre der wichtigsten Augenoperationen.

[82] Jüngken JC (1836). Die Lehre von den Augenkrankheiten.

[83] Rust JN (1830). Theoretisch-praktisches Handbuch der Chirurgie, mit Einschluß der syphilitischen und Augen-Krankheiten; in alphabetischer Ordnung.

[84] Most GF: Enzyklopädie der gesammten medicinischen und chirurgischen Praxis mit Einschluss der Geburtshilfe und der Augenheilkunde. Leipzg 1834.

[85] Follin E (1859). Leçons sur l'exploration de l'œil et en particulier sur les applications de l'ophtalmoscope au diagnostic des maladies des yeux.

[86] Dixon J (1855). A guide to the practical study of diseases of the eye: with an outline of their medical and operative treatment.

[87] Dixon J (1866). A guide to the practical study of diseases of the eye: with an outline of their medical and operative treatment. From the third London edition.

[88] von Graefe A, Saemisch: Handbuch der gesammten Augenheilkunde. 1876.

[89] Pflüger E. (1878). Neuritis optica. Albrecht von Græfe's Archiv für Ophthalmologie.

[90] von Leyden E (1863). Die graue Degeneration der hinteren Rückenmarksstränge.

[91] von Leyden E: Klinik der Rückenmarkskrankheiten. Berlin: Hirschwald; 1874–1876.

[92] Jarius S, Wildemann B (2012). An early British case of neuromyelitis optica (1850). BMJ.

[93] Jarius S, Wildemann B (2011). An early case of neuromyelitis optica: on a forgotten report by Jacob Lockhart Clarke, FRS. Mult Scler.

[94] Jarius S, Wildemann B (2013). On the contribution of Thomas Clifford Allbutt, F.R.S., to the early history of neuromyelitis optica. J Neurol.

[95] Erb W (1878). Krankheiten des Rückenmarks und des verlängerten Marks.

[96] Steffan P (1879). Beitrag zur Lehre des Zusammenhanges der Erkrankungen der Sehnerven mit denen des Rückenmarkes. Bericht über die Versammlung der Ophthalmologischen Gesellschaft, Heidelberg.

[97] Erb W (1880). Ueber das Zusammenvorkommen von Neuritis optica und Myelitis subacuta. Archiv für Psychiatrie und Nervenkrankheiten.

[98] Gowers WR: Discussion on eye symptoms in diseases of the spinal cord. Ophthalmological Society, June 7th and 8th, 1883. Transactions of the Ophthalmologicl Society of the UK 1883, 3 (session 1882–1883):190–204.

[99] Gowers WR (1883). Address on eye symptoms in diseases of the spinal cord. Delivered before the Ophthalmological Society, June 7th, 1883. Lancet.

[100] Ruete CGT (1845). Lehrbuch der Ophthalmologie für Aerzte und Studierende (1st edition).

[101] Ruete CGT (1853). Lehrbuch der Ophthalmologie für Aerzte und Studierende (2nd edition).

[102] Desmarres LA (1847). Traité théorique et pratique des maladies des yeux.

[103] Tetzer M (1870). Compendium der Augenheilkunde nach weil. Dr. Max Tetzer's systematischen Vorträgen. Herausgegeben von J. Grünfeld.

[104] Jarius S, Wildemann B (2012). The case of the Marquis de Causan (1804): an early account of visual loss associated with spinal cord inflammation. J Neurol.

[105] Stellwag von Carion C: Lehrbuch der praktischen Augenheilkunde (2 vols.). Wien: Wilhelm Braumüller; 1861/1862.

[106] Allbutt TC (1871). On the use of the ophthalmoscope in diseases of the nervous system and of the kidneys; also in certain other general disorders.

[107] Gowers WR (1879). A manual and atlas of medical ophthalmoscopy.

[108] Bouchut E (1866). Du diagnostic des maladies du système nerveux par l’ophthalmoloscopie.

[109] Meyer È (1880). Traité pratique des maladies des yeux.

[110] Khayat A, Rathore MH (2008). Treponema pallidum. The Neurological Manifestations of Pediatric Infectious Diseases and Immunodeficiency Syndromes.

[111] Ravin JG (1999). The statesman, the artist, and the ophthalmologist: Clemenceau, Lautrec, and Meyer. Arch Ophthalmol.

[112] Leber T, Graefe A, Saemisch T (1877). (Ed.). Die Spinalamaurose (§268). Handbuch der Gesammten Augenheilkunde.

[113] Mackenzie W (1854). A Practical treatise on the diseases of the eye.

[114] Soelberg Wells J (1869). A Treatise on the Diseases of the Eye.

[115] Angell HC (1870). A treatise on diseases of the eye; for the use of general practitioners.

[116] Rutkow IM (1988). The history of surgery in the United States 1775–1900. Volume 1.

[117] Phillips J (1869). Ophthalmic surgery and treatment: with advice on the use and abuse of spectacles.

[118] Kremer L, Mealy M, Jacob A, Nakashima I, Cabre P, Bigi S, Paul F, Jarius S, Aktas O, Elsone L, Mutch K, Levy M, Takai Y, Collongues N, Banwell B, Fujihara K, de Seze J (2013). Brainstem manifestations in neuromyelitis optica: a multicenter study of 258 patients. Multiple Sclerosis Journal.

[119] Allbutt TC (1870). On the ophthalmoscopic signs of spinal disease. Lancet.

[120] Swanzy HR (1892). A handbook of the diseases of the eye and their treatment.

[121] Werner LE (1915). Swanzy's handbook of the diseases of the eye and their treatment.

[122] Chauvel J (1880). Névrite optique double avec myélite aiguë temporaire. (Séance du 4 Aout.). Bull et mém Soc de chir de Paris.

[123] Rumpf T (1881). Mittheilungen aus dem Gebiet der Neuropathologie und Elektrotherapie. I. Zur Wirkung des faradischen Pinsels bei einem Fall von Neuritis optica mit Myelitis transversa. Dtsch Mediz Wochenschr.

[124] Noyes HD (1881). Acute myelitis mit doppelseitiger Neuritis optica. Archiv für Augenheilkunde.

[125] Dreschfeld J (1882). On two cases of acute myelitis associated with optic neuritis. Lancet.

[126] Peltesohn N (1886). Ursachen und Verlauf der Sehnervenatrophie. Zentralblatt für praktische Augenheilkunde.

[127] Peltesohn N (1886). Ursachen und Verlauf der Sehnervenatrophie. Zentralblatt für praktische Augenheilkunde.

[128] Peltesohn N (1886). Ursachen und Verlauf der Sehnervenatrophie. Zentralblatt für praktische Augenheilkunde.

[129] Achard C, Guinon L (1889). Sur un cas de myélite aiguë diffuse avec double névrite optique. Archives de medecine expérimentale et d'anatomie pathologique.

[130] Schanz F (1893). Ueber das Zusammenvorkommen von Neuritis optica und Myelitis acuta. Dtsch Med Wochenschr.

[131] Jarius S, Wuthenow AB, Wildemann B (2018). The first Japanese report on neuromyelitis optica rediscovered: acute bilateral blindness, tetraparesis and respiratory insufficiency in a 35-year-old man (1891). J Neurol Sci.

[132] Aoyama T (1891). A case of acute myelitis with simultaneous amaurosis – clinical study. Tôkyô igakkai zasshi (Journal der Tôkyô Medical Association).

[133] Devic E (1895). Myélite aiguë dorso-lombaire avec névrite optique. - Autopsie. Congrès français de médecine (Premiere Session; Lyon, 1894; Procès-verbaux, mémoires et discussions; Publiés par M le Dr L Bard).

[134] Dálen A (1899). Neuritis optica und myelitis acuta. Albrecht von Graefe's Archiv für Ophthalmologie.

[135] Jarius S, Wildemann B (2017). Devic's disease before Devic: On the contribution of Friedrich Albin Schanz (1863–1923). J Neurol Sci.

[136] Katz K (1896). Über das Zusammenvorkommen von Neuritis optica und Myelitis acuta.

[137] Devic E (1894). Myélite subaiguë compliquée de névrite optique. Le Bulletin Médicale.

[138] Jarius S., Wildemann B. (2019). Devic's index case: A critical reappraisal – AQP4-IgG-mediated neuromyelitis optica spectrum disorder, or rather MOG encephalomyelitis?. Journal of the Neurological Sciences.

[139] von Leyden E, Goldschneider J (1897). Die Erkrankungen des Rückenmarkes und der Medulla oblongata.

[140] Elschnig A (1893). Klinischer und anatomischer Beitrag zur Kenntnis der acuten retrobulbären Neuritis. Archiv für Augenheilkunde.

[141] Bielschowsky M (1901). Myelitis und Sehnerventzündung.

[142] Trnka von Krzowitz W (1790). Geschichte des schwarzen Staares in welcher die Erfahrungen der Aerzte aller Zeiten enthalten sind.

[143] Trnka de Krzowitz W (1781). Historia amauroseos omnis aevi observata medica continens. Pars I.

[144] Beer JG (1791). Beobachtungen über verschiedene, vorzüglich aber über jene Augenkrankheiten, welche aus allgemeinen Krankheiten des Körpers entspringen, oder öfters mit denselben verbunden sind.

[145] Beer JG (1792). Lehre der Augenkrankheiten. Zweiter Theil von den innerlichen Krankheiten des Auges.

[146] Beer JG (1792). Lehre der Augenkrankheiten. Erster Theil von den äußerlichen Krankheiten des Auges.

[147] Beer GJ (1813). Lehre von den Augenkrankheiten. Band 1.

[148] Beer GJ (1817). Lehre von den Augenkrankheiten. Band 2.

[149] Richter AG (1795). Anfangsgründe der Wundarzneykunst. Dritter Band.

[150] Himly K (1801). Ophthalmologische Beobachtungen und Untersuchungen oder Beyträge zur richtigen Kenntniss und Behandlung der Augen im gesunden und kranken Zustande.

[151] Himly K (1804). Bemerkungen über die Hauptarten der Amblyopie und Amaurose. Ophthalmologische Bibliothek.

[152] Himly K (1806). Einführung in die Augenheilkunde.

[153] Himly K (1830). Einleitung zur Augenheilkunde, für seine Vorlesungen geschrieben.

[154] Wenzel J. Manuel de l'oculiste, ou dictionnaire ophthalmologique. Tome I. Paris Lavater; 1808.

[155] Kieser DG (1811). Über die Natur, Ursachen, Kennzeichen und Heilung des schwarzen Staars.

[156] Wardrope J (1808). Essays on the morbid anatomy of the human eye.

[157] Wardrope J (1818). Essays on the morbid anatomy of the human eye. Vol. II.

[158] Demours AP (1818). Traite des maladies des yeux. Vol. I-IV.

[159] Guillié S (1818). Nouvelles recherches sur la cataracte et la goutte sereine (2nd edition).

[160] Langenbeck CJM (1815). Reflexionen über die Natur, Ursachen und Heilung des schwarzen Staares. Neue Bibliothek für die Chirurgie und Ophthalmologie.

[161] Vetch J (1820). A practical treatise on the diseases of the eye. London.

[162] Travers B (1820). A synopsis of the diseases of the eye, and their treatment.

[163] Travers B (1825). A synopsis of the diseases of the eye, and their treatment (1st American edn. from the 3rd London edn.).

[164] Stratford SJ (1828). A manual of the anatomy, physiology, & diseases of the eye and its appendages.

[165] Lawrence W (1830). A treatise on the venereal diseases of the eye.

[166] Lawrence W (1833). A treatise on the diseases of the eye.

[167] Fischer JN (1832). Klinischer Unterricht in der Augenheilkunde.

[168] Beck KJ (1832). Handbuch der Augenheilkunde: zum Gebrauche bei seinen Vorlesungen.

[169] Andreae AW (1834). Grundriss der allgemeinen Augenheilkunde.

[170] Bessières G-L (1838). Nouvelles considérations sur les affections nerveuses de l'organe de la vue, confondues par les auteurs sous le nom générique d'amaurose.

[171] Middlemore R (1835). A treatise on the diseases of the eye and its appendages; in two volumes. London, Birmingham: Longman, Rees, Orme.

[172] Rognetta M (1839). Cours d'ophthalmologie, ou Traité complet des maladies de l'oeil.

[173] Weller CH (1819). Die Krankheiten des menschlichen Auges.

[174] Weller CH (1826). Die Krankheiten des menschlichen Auges (3rd edition).

[175] Leffler CT, Randolph J, Stackhouse R, Davenport B, Spetzler K (2012). Monteath's translation of Weller: an underappreciated trove of ophthalmology lexicon. Arch Ophthalmol.

[176] Weller CH: A Manual of the diseases of the human eye, for surgeons commencing practice. Translated from the German by G. C. Monteath, 2 vols. London; 1821.

[177] Weller CH (1828). Traité théorique et pratique des maladies des yeux. Trad. d. L. Allemand sur la 3. éd. par F. J. Riester. 2 vol.

[178] Weller CH (1832). Traité théorique et pratique des maladies des yeux. Trad. d. L. Allemand sur la 3. éd. par F. J. Riester. 2 vol.

[179] Schmalz KG (1816). Versuch einer medizinisch-chirurgischen Diagnostik in Tabellen, oder Erkenntniß und Unterscheidung der inneren und äußeren Krankheiten, mittelst Nebeneinanderstellung der ähnlichen Formen (3rd edition).

[180] Ollivier dA (1824). C. P.: Moelle épinière et de ses maladies (1st edn.).

[181] Abercrombie J (1819). Observations on Diseases of the Spinal Marrow. Am Med Recorder.

[182] Abercrombie J (1828). Pathological and Practical Researches on Diseases of the Brain and Spinal Cord.

[183] Ollivier dA (1827). C. P.: Moelle épinière et de ses maladies (2nd edn., 2 vol.).

[184] Ollivier dA (1837). C. P.: Moelle épinière et de ses maladies (3rd edn., 2 vol.).

[185] Meryon E (1863). Practical and Pathological Researches on the Various Forms of Paralysis. Brit Med J.

[186] Cooper S (1822). Dictionary of Practical Surgery by Samuel Cooper.

[187] Cooper S (1822). Dictionary of Practical Surgery by Samuel Cooper (from the 4^th^ London edition).

[188] Frick G (1823). A treatise on the diseases of the eye.

[189] Schmalz KG (1825). Versuch einer medizinisch-chirurgischen Diagnostik in Tabellen, oder Erkenntniß und Unterscheidung der inneren und äußeren Krankheiten, mittelst Nebeneinanderstellung der ähnlichen Formen (4th edition).

[190] Frick G, Welbank R (1826). A treatise on the diseases of the eye.

[191] Rosas A (1830). Handbuch der theoretischen und practischen Augenheilkunde (Band 2).

[192] Schmalz KG (1830). Versuch einer medizinisch-chirurgischen Diagnostik in Tabellen, oder Erkenntniß und Unterscheidung der inneren und äußeren Krankheiten, mittelst Nebeneinanderstellung der ähnlichen Formen (4th edition).

[193] Cooper S (1836). Dictionary of Practical Surgery by Samuel Cooper.

[194] Lawrence W, Hays I (1854). A treatise on the diseases of the eye.

[195] Cooper S (1854). Dictionary of Practical Surgery by Samuel Cooper (from the 7^th^ edition).

[196] Wharton Jones T (1869). Failure of sight from railway and other injuries of the spine and head. With a physiological and pathological disquisition into the influence of the vaso-motor nerves on the circulation of the blood in the extreme vessels.

[197] Mooren A (1874). Ophthalmologische Mittheilungen aus dem Jahre 1873.

[198] Mooren A (1882). Fünf Lustren ophthalmologischer Thätigkeit.

[199] Oglesby RP (1874). Case of sclerosis of the optic discs following spinal injury. Br Med J.

[200] Thorowgood JC (1875). Case of optic neuritis, with complete loss of Vision. Recovery under treatment. Read January 22, 1875. Trans Clin Soc London.

[201] Thorowgood JC (1875). Case of optic neuritis, with complete loss of Vision. Recovery under treatment. Med Times Gazette.

[202] Erichsen JE (1875). On concussion of the spine. Nervous shock and other obscure injuries of the nervous system.

[203] Duplay S, Follin E (1875). Traité élémentaire de pathologie externe. Tome 3.

[204] Meyer È (1879). Handbuch der Augenheilkunde.

[205] Clarce B (1881). St. Bartholemews Hospital Reports 1880. London Med Record.

[206] Firth RH (1886). Double optic neuritis with paralysis of one arm following an injury to the spine. Practitioner.

[207] Taylor J (1901). Optic neuritis in diseases of the spinal cord. Injury, tumour, myelitis. (An account of twelve cases and one autopsy.). Brain.

[208] Baillie M (1793). Morbid anatomy of some of the most important parts of the human body.

[209] Rodin AE (Ed.). The influence of Matthew Baillie's Morbid anatomy; biography, evaluation, and reprint. Springfield, Ill.: Charles C. Thomas; 1973.

[210] Wardrope J (Ed.). The Works of Matthew Baillie, M.D.: To which is Prefixed an Account of His Life. London: Longman, Hurst, Rees, Orme, Brown, and Green; 1825.

[211] Macmichael W (1827). The gold-headed cane.

[212] Baillie M (1820). Some observations upon paraplegia in adults. Medical transactions, published by the London College of Physicians.

[213] Anonymous: Review VI. Lectures on the Diagnosis and Treatment of the Principal Forms of Paralysis of the Lower Extremities. By C. E. Brown Séquard, M.D. F.R.S., Physician to the National Hospital for the Paralysed and the Epileptic, &c. &c.–1861. Course of Lectures on the Physiology and Pathology of the Nervous System. Delivered at the Royal College of Surgeons of England. May, 1858. By C. E. Brown-Séquard, M.D., &c.–1860. British and Foreign Medico-chirurgical Review. Vol. XXIX, no. LVII, April 1862, p. 371–411.

[214] Baillie M, Wardrope J (1825). Additional observations on paraplegia in adults. The Works of Matthew Baillie, MD. Volume 1.

[215] Stephen L, Lee S. Dictionary of national biography. London: Macmillan; 1890.

[216] Munk K. The roll of the Royal College of Physicians of London. Vol. III (1801-1825). London: The College; 1878.

[217] Harrison Burder T (1827). An inquiry into the alleged cerebral origin of certain cases of paraplegia. London Med Physical J.

[218] Elliotson J (1830). Paraplegia. Lancet.

[219] Earle H (1827). On paraplegia (Read June 19, 1827). Medico-chirurgical Transactions, published by the Medical and Chirurgical Society of London.

[220] Anonymous (1834). Reports of the Westminster Medical Society. Paraplegia without disease of the spine. London Med Surg J.

[221] Medical Transactions of the College of Physicians in London (1821). Medico-Chirurgical Review J Med Sci Analytical Series.

[222] Anonymous (1829). Palsy of the lower limbs, (paraplegia). Monthly Gazette Health.

[223] Merton RK (1993). On the shoulders of giants : a Shandean postscript. Post-Italianate edn.

[224] Hennemann A (1845). Die differentielle medizinische Diagnostik, mit Einschluß der Hautkrankheiten.

[225] Pescetto GB. Storia di un caso di noteomielite acuta, accompagnata da amaurosi. Giornale delle Scienze Mediche della Societa Medico-Chirurgica di Torino. 1844:311–24.

[226] Maletius HJ (1837). Observationes nonnullae de myelitide institutae; dissertatio inauguralis.

[227] Friedrich GF (1825). De myelitide.

[228] Ware J: An enquiry into the causes which have most commonly prevented success in the operation of extracting the cataract; with an account of the means by which thy may either be avoided or rectified; to which are added oberservations on the dissipation of the cataract, and on the cure of the gutta serena. London: C. Dilly, H. Murray and J. Walter; 1795.

[229] Jarius S, Jacobi C, de Seze J, Zephir H, Paul F, Franciotta D, Rommer P, Mader S, Kleiter I, Reindl M (2011). Frequency and syndrome specificity of antibodies to aquaporin-4 in neurological patients with rheumatic disorders. Mult Scler.

[230] Power D (1899). Ware, James (1756–1815). In Dictionary of National Biography. Volume 59.

[231] Pettigrew TJ (1839). James Ware, F.R.S. In Biographical memoirs of the most celebrated physicians, surgeons, etc etc who have contributed to the advancement of medical science.

[232] Dunn P (1917). British Masters of Ophthalmology series. 3. - James Ware, F.R.S. (1756–1815). Brit J Ophthalmol.

[233] Voigtel FG (1816). Vollständigs System der Arzneymittellehre. Herausgegeben von Carl Gottlob Kühn.

[234] Bischoff CHE (1801). Commentatio de usu galvanismi in arte medica speciatim vero in morbis nervorum paralyticis.

[235] Warner J (1773). A description of the human eye, and its adjacent parts; together with their principal diseases, and the methods proposed for relieving them.

[236] Power D: Warner, Josef. In Dictionary of National Biography. Volume 59. Edited by Leslie S, Lee S. London Smith, Elder, and Co.; 1885–1900: 396–397.

[237] Argyll Robertson DMCL (1869). Article IV. - On an interesting series of eye-symptoms in a case of spinal disease, with remarks on the action of belladonna on the iris, etc. Edin Med J.

[238] Argyll Robertson DMCL (1869). Four cases of spinal miosis; with remarks on the action of light on the pupil. Edin Med J.

[239] Romberg MH (1840). Lehrbch der Nervenkrankheiten des Menschen, 1. Band, 2. Abteilung, p. 797.

[240] Romberg MH (1853). Manual of the Nervous Diseases of Men (2 vol.).

[241] Trousseau A (1865). Clinique médicale de L'Hotel-Dieu dè Paris. Tome deuxième. p. 512.

[242] Trousseau A (1867). Lectures on clinical medicine, delivered at the Hôtel-Dieu, Paris, tr. and ed. by P.V. Bazire, vol. I.

[243] Pearce JM (2004). The Argyll Robertson pupil. J Neurol Neurosurg Psychiatry.

[244] Leake CD (1970). Douglas Argyll Robertson. In The Founders of Neurology 2nd ed Edited by Haymaker W SF.

[245] Lovera L, Jay WM, Biller J (2014). Horner Syndrome in a Case of Neuromyelitis Optica. Neuroophthalmology.

[246] Tyrer JH, Sutherland JM, Eadie MJ (1963). Bilateral Miosis in Voluntary Deviations of the Eyes in All Directions, in a Syndrome of Optic Neuromyelitis. Rev Neurol (Paris).

[247] Ozcelik P, Tanriverdizade T, Men S, Akdal G (2017). Convergence spasm due to aquaporin-positive neuromyelitis optica spectrum disorder. eNeurologicalSci.

[248] Takahashi Y, Manabe Y, Nakano Y, Yunoki T, Kono S, Narai H, Omori N, Abe K (2016). Fulminant case of neuromyelitis optica spectrum disorder initiated with area postrema symptoms. Neurol Clin Neurosci.

[249] Uludag IF, Sariteke A, Ocek L, Zorlu Y, Sener U, Tokucoglu F, Uludag B (2017). Neuromyelitis optica presenting with horner syndrome: A case report and review of literature. Mult Scler Relat Disord.

[250] Wingerchuk DM, Banwell B, Bennett JL, Cabre P, Carroll W, Chitnis T, de Seze J, Fujihara K, Greenberg B, Jacob A (2015). International consensus diagnostic criteria for neuromyelitis optica spectrum disorders. Neurology.

[251] Erb W (1902). On spastic and syphilitic paralysis. Lancet.

[252] Wilcox RA, Burrow J, Slee M, Craig J, Thyagarajan D (2008). Neuromyelitis optica (Devic's disease) in a patient with syphilis. Mult Scler.

[253] Vidal Marsal F, Garcia Saavedra V, Gonzalez J, Richart Jurado C (1989). Devic's syndrome during secondary syphilis. An Med Interna.

[254] Alexander J (1827). On intermitting paraplegia, combined with amaurosis. Lancet.

[255] Malherbe. Notices sur le docteur Marcé. Annales de la sociéte académique de Nantes et du départment de la Loire-inferieure. 1860:31.

[256] Bradley J (1818). Observations on a stridulous affection of the bowels; and on some varieties of spinal disease; with an appendix of cases.

[257] Baumès JBT (1802). Fondemens de la science méthodique des maladies. Tome quatrième.

[258] Baumès JBT. Traité élémentaire de nosologie. Paris: Chez Crochart; 1806.

[259] Cullen W (1800). Nosology; or, a systematic arrangement of diseases, by classes, orders, genera, and species.

[260] Kraus LA (1831). Kritisch-etymologisches medicinisches Lexikon.

[261] von Grossi E (1831). Familiarum morborum humanorum expositio. Stuttgart, Tuebingen.

[262] Boisseau FG (1830). Nosographie organique. Tome quatrieme.

[263] Dunglison R (1848). Medical lexicon. A dictionary of medical science (7th edition).

[264] Piorry PA (1833). Mémoire sur les névralgies et sur leur traitement. Gaz méd.

[265] Piorry PA (1833). Clinique médicale de l'Hôpital de la Pitié : (Service de la Faculté de Médecine) et de l'Hospice de la Salpétrière en 1832.

[266] Piorry PA (1833). Mémoire sur les névralgies et sur leur traitement. Gaz méd.

[267] Piorry PA (1834). Remarks upon the Nature of Neuralgias, and their Treatment. Am J Med Sci.

[268] Piorry PA: Remarks upon the Nature of Neuralgias, and their Treatment. The Western Journal of the Medical and Physical Sciences 1835, Second hexade, Vol. II (Vol. VIII):130–137.

[269] Piorry PA (1850). Traité de médecine pratique et de pathologie iatrique ou médicale.

[270] Mongellaz PJ (1839). Monographie des irritations intermittentes.

[271] Casas de Mendoza N, Sampedro G (1843). Tratado completo de veterinaria (tomo II).

[272] Lanzillotti-Buonsanti N, Pini G (1875). Dizionario delle science mediche e veterinarie.

[273] Allbutt TC (1882). An address on the surgical aids of medicine. Brit Med J.

[274] Baker AB, Baker LH (1979). Clinical neurology.

[275] Fournier A (1875). De l'ataxie locomotrice d'origine syphilitique. Annales de Dermatologie et de Syphiligraphie.

[276] Fournier A (1876). De l’ataxie locomotrice d’origine syphilitique.

[277] Erb W (1879). Zur Pathologie der Tabes dorsalis. Deutsches Archiv für klinische Medizin.

[278] Vulpian A (1879). Maladies du système nerveux : leçons professées à la Faculté de médecine. Maladies de la moëlle / par A. Vulpian,... ; recueillies et publiées par M. le Dr Bourceret,... revues par le professeur.

[279] Gowers WR (1881). Syphilis and locomotor ataxy. Lancet.

[280] Hare EH (1959). The origin and spread of dementia paralytica. J Ment Sci.

[281] Szreter S (2014). The Prevalence of Syphilis in England and Wales on the Eve of the Great War: Re-visiting the Estimates of the Royal Commission on Venereal Diseases 1913–1916. Soc Hist Med.

[282] Anonymous (1903). Acute optic neuromyelitis. Brit Med J.

[283] Anonymous (1903). Acute optic neuromyelitis. Ophthlamoscope.

[284] Stransky E: 22) Neuromyèlite optique aiguë, par E. Brissaud et Brécy. (Review neurologique. 1904. Nr. 2.). Neurologisches Centralblatt 1904, 23:823–824.

[285] Acchiote P (1907). Sur un cas de neuromyélite subaiguë ou maladie de Devic. Bulletin officiels de la Société de neurologie de Paris.

[286] Acchiote P: Sur un cas de neuromyélite subaiguë ou maladie de Devic. Annales d'oculistique 1907, 70th year:374.

[287] Acchiote P (1907). Sur un cas de neuromyélite subaiguë ou maladie de Devic. Revue Neurologique.

[288] Matiello M, Schaefer-Klein J, Sun D, Weinshenker BG (2013). Aquaporin 4 expression and tissue susceptibility to neuromyelitis optica. JAMA Neurol.

[289] Tso MO, Shih CY, McLean IW (1975). Is there a blood-brain barrier at the optic nerve head?. Arch Ophthalmol.

[290] Hofman P, Hoyng P, vanderWerf F, Vrensen GF, Schlingemann RO (2001). Lack of blood-brain barrier properties in microvessels of the prelaminar optic nerve head. Invest Ophthalmol Vis Sci.

[291] Huang XN, Wang WZ, Fu J, Wang HB (2011). The relationship between aquaporin-4 expression and blood-brain and spinal cord barrier permeability following experimental autoimmune encephalomyelitis in the rat. Anat Rec (Hoboken).

[292] Wang Y, Zhu M, Liu C, Han J, Lang W, Gao Y, Lu C, Wang S, Hou S, Zheng N (2018). Blood Brain Barrier Permeability Could Be a Biomarker to Predict Severity of Neuromyelitis Optica Spectrum Disorders: A Retrospective Analysis. Front Neurol.

[293] Wilhelm I, Nyul-Toth A, Suciu M, Hermenean A, Krizbai IA (2016). Heterogeneity of the blood-brain barrier. Tissue Barriers.

[294] Jarius S, Wandinger KP, Borowski K, Stoecker W, Wildemann B (2012). Antibodies to CV2/CRMP5 in neuromyelitis optica-like disease: case report and review of the literature. Clin Neurol Neurosurg.

[295] Thompson AJ, Banwell BL, Barkhof F, Carroll WM, Coetzee T, Comi G, Correale J, Fazekas F, Filippi M, Freedman MS (2018). Diagnosis of multiple sclerosis: 2017 revisions of the McDonald criteria. Lancet Neurol.

[296] Marignier R, Bernard-Valnet R, Giraudon P, Collongues N, Papeix C, Zephir H, Cavillon G, Rogemond V, Casey R, Frangoulis B (2013). Aquaporin-4 antibody-negative neuromyelitis optica: distinct assay sensitivity-dependent entity. Neurology.

[297] Marignier R, Cobo Calvo A, Vukusic S (2017). Neuromyelitis optica and neuromyelitis optica spectrum disorders. Curr Opin Neurol.

[298] Marignier R, De Seze J, Vukusic S, Durand-Dubief F, Zephir H, Vermersch P, Cabre P, Cavillon G, Honnorat J, Confavreux C (2008). NMO-IgG and Devic's neuromyelitis optica: a French experience. Mult Scler.

[299] Marignier R, Giraudon P, Vukusic S, Confavreux C, Honnorat J (2010). Anti-aquaporin-4 antibodies in Devic's neuromyelitis optica: therapeutic implications. Ther Adv Neurol Disord.

[300] Marignier R, Nicolle A, Watrin C, Touret M, Cavagna S, Varrin-Doyer M, Cavillon G, Rogemond V, Confavreux C, Honnorat J, Giraudon P (2010). Oligodendrocytes are damaged by neuromyelitis optica immunoglobulin G via astrocyte injury. Brain.

[301] Marignier R, Ruiz A, Cavagna S, Nicole A, Watrin C, Touret M, Parrot S, Malleret G, Peyron C, Benetollo C (2016). Neuromyelitis optica study model based on chronic infusion of autoantibodies in rat cerebrospinal fluid. J Neuroinflammation.

[302] Weinshenker Brian G, Vukusic Sandra, Clanet Michel G (2013). Christian Confavreux (1949 – 2013). Multiple Sclerosis Journal.

[303] Hirsch A, Wernich A (1885). Biographisches Lexikon der hervorragenden Aerzte aller Zeiten und Völker. Band 2.

[304] Czerny V (1894). Rede des Herrn Geh. Rath Prof. Dr. Czerny, gehalten bei der Eröffnung des neuen Operationssaales der akademischen Klinik in Heidelberg am 15. Juli 1894. Ärztliche Mitteilungen aus und für Baden.

[305] Knapp H (1885). Ein Fall von acuter Myelitis mit beiderseitiger Ophthalmologie und Stauungspapille. Berliner Klinische Wochenschrift.

[306] Jarius S, Wildemann B (2013). Aquaporin-4 antibodies (NMO-IgG) as a serological marker of neuromyelitis optica: a critical review of the literature. Brain Pathol.

[307] Jarius S, Probst C, Borowski K, Franciotta D, Wildemann B, Stoecker W, Wandinger KP (2010). Standardized method for the detection of antibodies to aquaporin-4 based on a highly sensitive immunofluorescence assay employing recombinant target antigen. J Neurol Sci.

[308] Jarius S, Paul F, Franciotta D, Ruprecht K, Ringelstein M, Bergamaschi R, Rommer P, Kleiter I, Stich O, Reuss R (2011). Cerebrospinal fluid findings in aquaporin-4 antibody positive neuromyelitis optica: results from 211 lumbar punctures. J Neurol Sci.

[309] Jarius S, Franciotta D, Paul F, Ruprecht K, Bergamaschi R, Rommer PS, Reuss R, Probst C, Kristoferitsch W, Wandinger KP, Wildemann B (2010). Cerebrospinal fluid antibodies to aquaporin-4 in neuromyelitis optica and related disorders: frequency, origin, and diagnostic relevance. J Neuroinflammation.

